# Metingear: a development environment for annotating genome-scale metabolic models

**DOI:** 10.1093/bioinformatics/btt342

**Published:** 2013-06-13

**Authors:** John W. May, A. Gordon James, Christoph Steinbeck

**Affiliations:** ^1^Cheminformatics and Metabolism, European Molecular Biology Laboratory - European Bioinformatics Institute (EMBL-EBI), Wellcome Trust Genome Campus, Hinxton, Cambridge, CB10 1SD, UK and ^2^Unilever Discover, Colworth Science Park, Bedford, MK44 1LQ, UK

## Abstract

**Summary:** Genome-scale metabolic models often lack annotations that would allow them to be used for further analysis. Previous efforts have focused on associating metabolites in the model with a cross reference, but this can be problematic if the reference is not freely available, multiple resources are used or the metabolite is added from a literature review. Associating each metabolite with chemical structure provides unambiguous identification of the components and a more detailed view of the metabolism. We have developed an open-source desktop application that simplifies the process of adding database cross references and chemical structures to genome-scale metabolic models. Annotated models can be exported to the Systems Biology Markup Language open interchange format.

**Availability:** Source code, binaries, documentation and tutorials are freely available at http://johnmay.github.com/metingear. The application is implemented in Java with bundles available for MS Windows and Macintosh OS X.

**Contact:** johnmay@ebi.ac.uk

**Supplementary information:** Supplementary data are available at *Bioinformatics* online.

## 1 INTRODUCTION

Genome-scale metabolic models have proved useful in areas such as growth optimization and metabolic engineering (Thiele and Palsson, 2010). The time required to generate a reconstruction has decreased with improvements in automatic pipelines, such as the popular model-SEED (Henry *et al.*, 2010). Despite rapid improvements in automation, extensive manual curation is still required for an accurate model. Common practice is to download the draft reconstruction and curate the model in a text-based spreadsheet, adding missing reactions from multiple databases and literature reviews. Although recent models are also published in Systems Biology Markup Language (SBML) (Hucka *et al.*, 2010), the spreadsheets contain additional annotations that are not transferred to the SBML annotation model. The main advantage of the annotations is that they allow identification of the components in a model. Identifying which components are present allows it to be integrated and analyzed with other resources, such as metabolomics experiments, without additional effort to map the components between datasets.

The large number of components in genome-scale models means comprehensive annotation is time-consuming. For metabolites, these annotations constitute a cross reference to a chemical or a metabolic pathway database. Although this can help to identify what the metabolite is, there are limitations: (i) if the metabolite was added from a literature review, the reference dataset may not contain that entity, (ii) the reference dataset contains the entity but at a different protonation state (the protonation state of the entry is important, as conservation of mass and charge is a requirement for constraint-based analysis) and (iii) the reference databases are closed access or no longer fully accessible. The Kyoto Encyclopedia of Genes and Genomes (KEGG) (Kanehisa *et al.*, 2012) is frequently used as a starting point for models, but a paid subscription is now required for bulk access.

Chemical structure is already used in smaller models of metabolic pathways where atom–atom mappings between reactions are required for metabolic flux analysis. The inclusion of full chemical structure in a genome-scale metabolic model is advantageous over only including a cross reference. The structure is database independent and allows direct metabolite identification. A reference to any database can be inferred by searching for an isomorphic structure. Identification is required when incorporating several reference datasets. If no mapping exists, then additional processing is required to identify similarities and overlap. Integrating the chemical structure directly into the model facilitates this integration on demand. Reconciled databases (see Reconciled Databases, Supplementary Material) primarily use a structure representation to merge entities between datasets. These requirements are most beneficial as models grow larger. The recent human reconstruction (Thiele *et al.*, 2013) includes IUPAC International Chemical Identifier (InChI) structure representations. The structure also allows identification of metabolites that cannot be referenced to an existing database. A metabolite may be added from a literature review or identified experimentally from a mass spectrum. Models may include small peptide chains or fatty acids associated with the acyl carrier protein. These metabolites are not normally found in chemical databases but can still be assigned a chemical structure. Subtleties of metabolites and reactions can be expressed and interpreted from the structure. Metabolites are often referred by their trivial name, from which it is difficult to determine such subtleties. In particular, the full structure can unambiguously describe the protonation state and the stereochemistry.

Thermodynamic constraints and Gibbs free energy of formation can be estimated and used to constrain a model (Jankowski *et al.*, 2008). To estimate these constraints, the chemical structure is required.

We have created Metingear, a desktop application to assist in the annotation of metabolic models using both manual and automated techniques. The main goal is to facilitate the integration of chemical structure into genome-scale models.

## 2 EXISTING SOFTWARE

Desktop tools that can specifically provide annotation of model components include Pathway Tools (Karp *et al.*, 2010), the SuBliMinaL Toolbox (Swainston *et al.*, 2011), Metannogen (Gille *et al.*, 2011) and GEnome-scale Metabolic models Simulation, Reconstruction and Visualization (GEMSiRV) (Liao *et al.*, 2012).

Pathway Tools provides a comprehensive framework for creating and querying models. Components can be annotated with a cross reference to multiple databases, but these cannot be exported to SBML. Chemical structure can be included with metabolites, but it must be added manually for each entry.

The SuBliMinaL Toolbox allows the import of models from KEGG, MetaCyc (Karp *et al.*, 2010) and SBML. The toolbox can annotate compartments, metabolites and enzymes and merge models together using normalized annotations to the Chemical Entities of Biological Interest (ChEBI) ontology (Hastings *et al.*, 2013). The metabolite name is used to find candidate cross references, which are then added silently or selected from a list of suggestions.

Metannogen allows manual editing of annotations in SBML files locally and via a group annotation server; no assistance is provided for creating new annotations.

GEMSiRV provides construction, simulation and visualization of metabolic models. GEMSiRV allows import of models from a spreadsheet, provided the required columns are in the correct order. Annotations are fixed for each metabolic entity only allowing cross referencing to two specific resources. Annotations are neither automated nor exported to SBML.

## 3 KEY FEATURES

Metingear can import existing models from SBML, KEGG Markup Language (http://www.kegg.jp/kegg/xml/) and Microsoft Excel spreadsheets. Genes, gene products and annotations from a partial or fully assembled genome can also be imported (see Additional Features, Supplementary Material).

Metingear provides a dynamic annotation system (Supplementary Table S1) allowing each entity to hold multiple annotations of the same type. The application allows annotation of multiple cross references and is not coupled to a specific resource like other tools. If available, each new cross reference is automatically encoded with Minimum Information Required in the Annotation of Models (MIRIAM) registry (Juty *et al.*, 2012) information, which semantically describes the cross reference allowing interoperability with other tools. The resource to which a reference is referring can be inferred for manually entered identifiers, improving usability when adding cross references. Reactions can be entered via a reaction equation, and individual reaction participants can be added, modified and removed allowing for the representation of novel reactions.

**Fig. 1.**
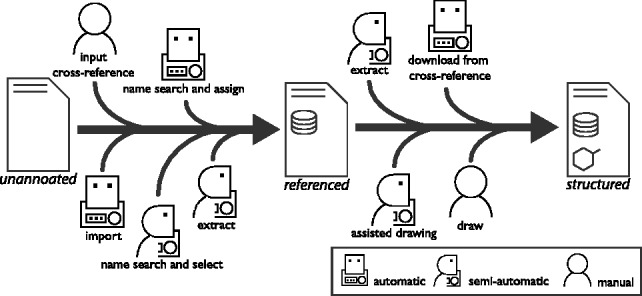
Overview of the annotation procedures to attach a chemical structure. Each method indicates whether the process is entirely automated, requires manual user input or combines an automated process with minimal user input

Internally, Metingear uses the Chemistry Development Kit (Steinbeck *et al.*, 2003) to represent and manipulate chemical structure. Multiple methods are used to attain a metabolite that is annotated with a chemical structure (Fig. 1). If a cross reference is available, a local or web service can be used to attach the structure. To improve performance, Metingear can create a local searchable index for data from multiple resources (see Services, Supplementary Material). Cross references can be imported directly from annotated SBML annotations or spreadsheet columns. Annotations, including cross references, can be extracted from notes provided by SBML input; recent models may provide a chemical structure in line notations (International Chemical Identifier), which can also be transferred. If there are no cross references available for import, a name search can be used to find a reference from a user-selected resource. As in the SuBliMinaL Toolbox, entries can be silently assigned if there is no difference in name or selected from a ranked list of candidates. If no cross reference can be found, the structure can be attached manually from several file formats or created semiautomatically. Dipeptides are often found in reconstructions that model peptidoglycan synthesis, but a chemical structure database may only provide entries for the individual residues. Dipeptide and polypeptide structures can be automatically created by matching the metabolite name.

Errors and inconsistency checks are provided to ensure a robust model. Annotation of several published models identified some missing information that could not be found in the native spreadsheets (see Inconsistencies, Supplementary Material).

## 4 FUTURE DIRECTIONS

The chemical structure can be used to merge metabolites and reactions in reconciled databases. Metingear allows multiple model formats to be annotated and standardized for creation of custom datasets and analysis. Future directions include extending the assisted structure drawing to uniquely represent more metabolites, such as acyl carrier protein-associated fatty acids. Substructure searching and tools to compare the chemical diversity in and between metabolic models will also be integrated. With many reconstruction pipelines already available, future work will primarily focus on model analysis and bridging the chemical gap between manual curation and the draft reconstruction.

*Funding*: Biotechnology and Biological Sciences Research Council CASE studentship [BB/I532153/1].

*Conflict of Interest*: none declared.

## Supplementary Material

Supplementary Data
