# Supplementary material for: Metingear: a development environment for annotating genome-scale metabolic models
Source: Bioinformatics. 2013 Jun 13;29(17):2213–5. doi: 10.1093/bioinformatics/btt342 (PMC3740624; doi:10.1093/bioinformatics/btt342)
Supplement: Supplementary Data [file supp_29_17_2213__index.html]

Metingear: A development environment for annotating genome-scale metabolic models — Metingear: a development environment for annotating genome-scale metabolic models — Metingear: a development environment for annotating genome-scale metabolic models — Supplementary Data 

# Metingear: a development environment for annotating genome-scale metabolic models

## 

files

**Files in this Data Supplement:**

- Supplementary Data - pdf file
- Supplementary Data - pdf file
