# Supplementary material for: Metingear: a development environment for annotating genome-scale metabolic models
Source: Bioinformatics. 2013 Jun 13;29(17):2213–5. doi: 10.1093/bioinformatics/btt342 (PMC3740624; doi:10.1093/bioinformatics/btt342)
Supplement: Supplementary Data [file supp_btt342_supplementry-unmarked.pdf]

Supplementary Material

April 17, 2013

Reconciled Databases Recently there has been an emergence of aggregated databases that reconcile entries from multiple *child* databases into a single *parent* database. Databases such as BKM-React (Lang *et al.*, 2011), MetRxn (Kumar *et al.*, 2012) and MNXref (Bernard *et al.*, 2012) offer an important resource to access metabolic and enzymatic information. However, these aggregated databases are limited by the substantial effort required to keep up to date with their respective *child* databases and license restrictions on redistribution. Generally chemical structure line notations, SMILES and InChI (Warr, 2011), are used to reconcile metabolites in these resources. How metabolites and reactions are merged can vary, one resource may merge entities at different protonation state whilst others may kept them separate. A molecule with unspecified stereo-chemistry may be merged with one which is fully specified, or different stereo isomers may all be collapsed to a generic entry.

Chemical File Formats Metingear can load from several file formats including, Chemical Markup Language (Kuhn *et al.*, 2007), Mol (Warr, 2011), The IUPAC International Chemical Identifier (InChI) and SMILES (Warr, 2011). Models can be exported in SBML with annotations of cross-references and InChI. Mol and CML files provide the exact depiction of what the original author of the structure drew whilst InChI and SMILES requires a new structure diagram is generated. Currently direct drawing of structures is not available. The JChemPaint (`http://jchempaint.github.com/`) (Krause *et al.*, 2000) application uses a incompatible version of the CDK library.

Services Metingear currently provides services to access; ChEBI (Matos *et al.*, 2010), MetaCyc (Caspi *et al.*, 2012), KEGG Compound (Kanehisa *et al.*, 2012), LIPID Maps (Sud *et al.*, 2007), Human Metabolome Database (HMDB) (Wishart *et al.*, 2009), PubChem-Compound (Bolton *et al.*, 2008) and UniProt (The UniProt Consortium, 2012). Each service, except PubChem, has a loader which allows the user to update the resource with the latest available version. When the download is small enough and available the resource can be updated automatically, in the cases of KEGG and MetaCyc where a fee or registration is required the file can be specified as a location on the local file system (see. `https://github.com/johnmay/metingear/wiki/Resources`). The loaders create a searchable index in the specified folder. The services then check for this index when a new tool is opened, if the index has been created then the service is available. As with the cross-references each service is linked with MIRIAM registry (Juty *et al.*, 2012) information. This allows Metingear to recognise which resource a metabolite is annotated with and try and locate a required service (e.g. name search, structure download). If there is no local index service loaded then it will default to a web-service query. The services load dynamically at runtime and thus it is possible to add custom services which may connect to in-house databases or web-services and provided specialised compounds. This feature also makes it very easy to integrate new resources (such as the reconciled databases) and keep up to date with the existing resources. Recently HMDB changed their download format, to accommodate this change, only a new loader for the format was required. The existing loader has been kept for legacy and still usable but the existing HMDB services access did not need to be changed.

Inconsistencies Annotating previously published models revealed inconsistencies which could not be easily identified in the original spreadsheet. A model of *Lactobacillus plantarum* WCFS1 (Teusink *et al.*, 2006) was found to be missing a reaction equation for reaction UGMDDS2 (Fig. S1). Also in a model of *Bacillus subtilis* (*i*Bsu1103) (Henry *et al.*, 2009), three reactions were found to reference a metabolite not found in the metabolites table (Fig. S2a and S2b). These inconsistencies demonstrate the use of specialised software in curation of larger reconstructions. These inconsistencies were identified automatically when a model is loaded from Excel. Other inconsistencies checks are carried out in the background of the model but do not declare an error. The mass and

charge balance of a reaction is indicated by *scales* icon which tips to which ever side is heavier or is balanced when the reaction is balanced. Structures attached to metabolites are checked as to whether they match encoded formulas and charges (which can be imported and extracted). This indication serves only as a hint that something might be wrong as the charge and formula annotations may be absent.

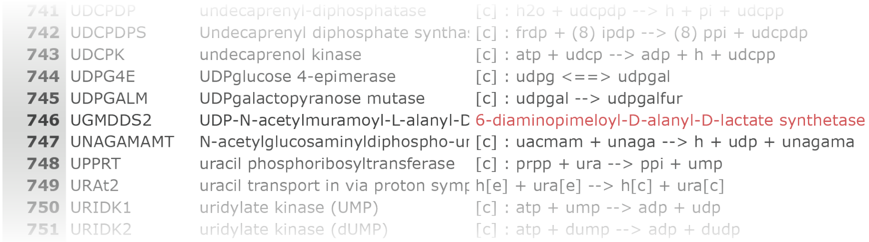

741	UDCPDP	undecaprenyl-diphosphatase	[c] : h2o + udcpdp --> h + pi + udcpp
742	UDCPDPS	Undecaprenyl diphosphate syntha:	[c] : frdp + (8) ipdp --> (8) ppi + udcpdp
743	UDCPK	undecaprenol kinase	[c] : atp + udcp --> adp + h + udcpp
744	UDPG4E	UDPglucose 4-epimerase	[c] : udpg <==> udpgal
745	UDPGALM	UDPgalactopyranose mutase	[c] : udpgal --> udpgalfur
746	UGMDDS2	UDP-N-acetylmuramoyl-L-alanyl-D	6-diaminopimeloyl-D-alanyl-D-lactate synthetase
747	UNAGAMAMT	N-acetylglucosaminyldiphospho-ur	[c] : uacmam + unaga --> h + udp + unagama
748	UPPRT	uracil phosphoribosyltransferase	[c] : prpp + ura --> ppi + ump
749	URAt2	uracil transport in via proton symp	h[e] + ura[e] --> h[c] + ura[c]
750	URIDK1	uridylate kinase (UMP)	[c] : atp + ump --> adp + udp
751	URIDK2	uridylate kinase (dUMP)	[c] : atp + dump --> adp + dudp

Figure 1: Subset of *Lactobacillus plantarum* WCFS1 reactions The reactions as listed in the the 'reaction info' sheet of 'Supplementary_material_IV.xls' of Teusink *et al.* (2006). The reaction in row 746 with the identifier `UGMDDS2` is missing a reaction equation and is instead replaced with the name of another reaction.

Additional Features In addition to handling metabolites and reactions their is also support for genes and gene products. These can be imported from the European Nucleotide Archive (ENA) XML (Amid *et al.*, 2012) and fasta formats. When importing models from a spreadsheet the locus of the reaction is often annotated. This locus annotation can be paired with the gene/gene product information to provide a model which is enriched with sequence as well as chemical information. Although not the primary purpose Metingear can also run a homology search using a locally installed BLAST (Altschul *et al.*, 1990) instance and transfer the annotations from homologous sequences. We are currently focused on metabolite annotation but in future we will improve the gene and gene product linking to be more automated.

A real-time search, undo/redo edit support, star rating and sub-collections help in general navigation. All entities can be easily renamed merged and split allowing the flexibility when editing a reconstruction. Each entity can have it's name and abbreviation changed the primary identifier assigned automatically. Each model is encoded with taxonomy information and when available compartments are annotated with Gene Ontology Terms (Camon, 2003) in the SBML output.

Each metabolite with a structure, molecular formula and charge indicates via *structural validity* whether the structure matches the given formula and charge. The formula and charge can often be imported from the spreadsheets or SBML notes and provides a check as to whether the attached structure is correct. Reactions indicate whether their participants are balanced (mass only) and whether they are transport reactions.

Internally a binary format is used for the reconstructions, this format provides very rapid loading and saving of reconstructions. Draft reconstructions from the model-SEED (Henry *et al.*, 2010) can be directly imported via the spreadsheet format without having to select which fields are present. Metingear can also create and export a stoichiometric matrix to a tabular file or to a '.sif' which can be loaded in Cytoscape (`http://www.cytoscape.org/`). The chemical structure of metabolites in the models can be exported to a single structured-data file (SDF) (Warr, 2011).

A primitive but functional dialog plugin framework allows one to extend Metingear with their own tools (`https://github.com/johnmay/metingear/wiki/Plugable-Dialogs`).

Table 1: Available annotations - each annotation can be added one or more times to a model component. Some annotations may have restrictions on where they can be added (e.g. flux annotations can only be added to reactions)

ACP Associated	Indicate a metabolite as being attached to an Acyl Carrier Protein
Lumped	Indicate that a metabolite or reactions is not a discrete entity and rather it is an average of many entities. It is common practise to include such metabolitesas DNA/RNA/Fatty Acid Composition in biomass reactions
Cross-reference	A cross-reference to an resource that describes this model component
Classification	A cross-reference that specifically links to a classification resource (e.g. GOTerm or E.C. Number)
Citation	A cross-reference that specifies a literature resource
ChEBI Cross-reference	Utility cross-reference specific to the ChEBI database
Enzyme Classification	Utility cross-reference to the Enzyme Classification (E.C.) number
KEGG Cross-reference	Utility cross-reference that specifically links to the KEGG Compound database
Chemical Structure	The chemical structure of the metabolite
InChI	The IUPAC International Chemical Identifier line notation of chemical structure
SMILES	The simplified molecular-input line-entry specification representation of chemical structure
Charge	The charge of this metabolite
Exact Mass	The exact mass is the sum of the masses of the atoms in a molecule using the most abundant isotope for each element
Molecular Formula	The chemical formula of a metabolite
Flux Bound	Bounding of metabolic flux
Flux Lower Bound	A lower bound for reaction flux
Flux Upper Bound	A upper bound for reaction flux
Gibbs Energy (ΔG)	Thermodynamic potential of the reaction
Comment	A short comment that has been added by an author
Note	A short note/comment about an entity which has no know author
Source	Non-semantic description of where the entity has come from
Subsystem	Abstract functional role of this reaction
Synonym	An alternative name for this entity
Systematic Name	A systematic name for an entity
Locus	The gene locus/association

622	rxn03164	UDP-N-acetylmuramoyl-L-alanyl-D	cpd00002 + cpd02964 + cpd00731 => cpd00008 + cpd(
623	rxn03167	2-Amino-4-hydroxy-6-(erythro-1,	3 cpd00001 + cpd02978 => 3 cpd00009 + 3 cpd00067 -
624	rxn03175	N-(5'-Phospho-D-ribosylforminminc	cpd02979 <=> cpd02991
625	rxn03194	(S)-2-Aceto-2-hydroxybutanoate	cpd03049 + cpd00094 <=> cpd00056 + cpd00498
626	rxn03239	(S)-3-Hydroxyhexadecanoyl-CoA:	cpd00003 + cpd03113 <=> cpd00067 + cpd00004 + cp
627	rxn03240	(S)-3-Hydroxyhexadecanoyl-CoA	cpd03113 <=> cpd00001 + cpd03126
628	rxn03241	(S)-3-Hydroxytetradecanoyl-CoA	cpd03115 <=> cpd00001 + cpd03127
642	rxn03409	Undecaprenyl-diphospho-N-acetyl	cpd00002 + cpd00013 + cpd03495 => cpd00008 + cpd(
643	rxn03424	L-erythro-4-Hydroxyglutamate:NA	2 cpd00067 + cpd00004 + cpd01974 <=> cpd00003 + (
644	rxn03435	(R)-2,3-Dihydroxy-3-methylpenta	cpd00006 + cpd02535 <=> cpd00067 + cpd00005 + cp
645	rxn03436	(S)-2-Aceto-2-hydroxybutanoate:	cpd00498 <=> cpd10162
646	rxn03437	(R)-2,3-Dihydroxy-3-methylpenta	cpd02535 => cpd00001 + cpd00508
647	rxn03445	O-Phospho-4-hydroxy-L-threonine	cpd00024 + cpd03607 <=> cpd00023 + cpd03606
648	rxn03481	Arbutin 6-phosphate glucohydrola	cpd00001 + cpd03697 <=> cpd00415 + cpd00863
1190	rxn08615	glycogen synthase (ADPGlc)	cpd00387 + cpd15302 <=> cpd00008 + cpd00155
1191	rxn08669	Glycerophosphodiester phosphodi	cpd00001 + cpd02090 <=> cpd00080 + cpd00100
1192	rxn08707	Heme O synthase|protoheme ix fa	cpd00001 + cpd00028 + cpd00350 <=> cpd00012 + cp
1193	rxn08764	ketol-acid reductoisomerase (2-Ac	cpd00067 + cpd00005 + cpd00498 <=> cpd00006 + cp
1194	rxn08775	L-alanyl-gamma-L-glutamate pept	cpd00001 + cpd15388 <=> cpd00023 + cpd00035
1195	rxn09011	nucleoside-triphosphatase (dITP)	cpd00001 + cpd00977 => cpd00009 + cpd00067 + cpd(
1196	rxn09012	nucleoside-triphosphatase (XTP)	cpd00001 + cpd00530 => cpd00009 + cpd00067 + cpd(

(a) subset of *Bacillus subtilis* (*i*Bsu1103) reactions

322	cpd00491	D-Mannitol 1-phosphate|D-mannit	C6H14O9P	C00644
323	cpd00492	N-Acetyl-D-mannosamine|2-Aceta	C8H15NO6	C00645
324	cpd00497	Xanthosine 5'-phosphate|Xanthyli	C10H12N4O9P	C00655
325	cpd00501	beta-D-Glucose 1-phosphate|beta	C6H12O9P	C00663
326	cpd00504	LL-2,6-Diaminoheptanedioate|LL-	C7H14N2O4	C00666
327	cpd00507	sn-glycero-3-Phosphocholine|Glyc	C8H20NO6P	C00670

(b) subset of *Bacillus subtilis* (*i*Bsu1103) metabolites

Figure 2: Missing information in genome-scale models.
2a) Three reactions `rxn003194`, `rxn03436` and `rxn08764` from the reactions spreadsheet (Table S2-Reaction Data) reference a metabolite, `cpd00498` (highlighted red), however information about this metabolite is missing from the metabolites sheet (Table S1-Compound Data) (Henry *et al.*, 2009).
2b) Expected location of the missing metabolite `cpd00498` in the metabolites table (marked with a red line). Using the named reaction (not shown) it is possible to see that the name of missing metabolite is 2-aceto-2-hydroxybutanoate but no other details to this metabolite are provided.

References

Altschul, S. F., Gish, W., Miller, W., Myers, E. W., and Lipman, D. J. (1990). Basic local alignment search tool. *J Mol Biol*, **215**(3), 403–10.

Amid, C., Birney, E., Bower, L., Cerdeno-Tarraga, A., Cheng, Y., Cleland, I., Faruque, N., Gibson, R., Goodgame, N., Hunter, C., Jang, M., Leinonen, R., Liu, X., Oisel, A., Pakseresht, N., Plaister, S., Radhakrishnan, R., Reddy, K., Riviere, S., Rossello, M., Senf, A., Smirnov, D., Hoopen, P. T., Vaughan, D., Vaughan, R., Zalunin, V., and Cochrane, G. (2012). Major submissions tool developments at the European Nucleotide Archive. *Nucleic Acids Research*, **40**(D1), D43–D47.

Bernard, T., Bridge, A., Morgat, A., Moretti, S., Xenarios, I., and Pagni, M. (2012). Reconciliation of metabolites and biochemical reactions for metabolic networks. *Briefings in Bioinformatics*, **pp**, 1–133.

Bolton, E., Wang, Y., Thiessen, P., and Bryant, S. (2008). Pubchem: Integrated platform of small molecules and biological activities. *Annual Reports in Computational Chemistry, American Chemical Society, Washington, DC*, **4**.

Camon, E. (2003). The gene ontology annotation (goa) project: Implementation of go in swiss-prot, trembl, and interpro. *Genome Research*, **13**(4), 662–672.

Caspi, R., Altman, T., Dreher, K., Fulcher, C. A., Subhraveti, P., Keseler, I. M., Kothari, A., Krummenacker, M., Latendresse, M., Mueller, L. A., Ong, Q., Paley, S., Pujar, A., Shearer, A. G., Travers, M., Weerasinghe, D., Zhang, P., and Karp, P. D. (2012). The MetaCyc database of metabolic pathways and enzymes and the BioCyc collection of Pathway/Genome Databases. *Nucleic Acids Research*, **40**(D1), D742–D753.

Henry, C. S., Zinner, J. F., Cohoon, M. P., and Stevens, R. L. (2009). *i*Bsu1103: a new genome-scale metabolic model of *Bacillus subtilis* based on SEED annotations. *Genome Biol*, **10**(6), R69.

Henry, C. S., DeJongh, M., Best, A. A., Frybarger, P. M., Linsay, B., and Stevens, R. L. (2010). High-throughput generation, optimization and analysis of genome-scale metabolic models. *Nature Biotechnology*, **28**(9), 969–974.

Juty, N., Novère, N. L., and Laibe, C. (2012). Identifiers.org and MIRIAM Registry: community resources to provide persistent identification. *Nucleic Acids Research*, **40**(D1), D580–D586.

Kanehisa, M., Goto, S., Sato, Y., Furumichi, M., and Tanabe, M. (2012). KEGG for integration and interpretation of large-scale molecular data sets. *Nucleic Acids Research*, **40**(D1), D109–D114.

Krause, S., Willighagen, E., and Steinbeck, C. (2000). Jchempaint - using the collaborative forces of the internet to develop a free editor for 2d chemical structures. *Molecules*, **5**(1), 93–98.

Kuhn, S., Helmus, T., Lancashire, R. J., Murray-Rust, P., Rzepa, H. S., Steinbeck, C., and Willighagen, E. L. (2007). Chemical Markup, XML, and the world wide web. 7. cmlspect, an xml vocabulary for spectral data. *J Chem Inf Model*, **47**(6), 2015–34.

Kumar, A., Suthers, P., and Maranas, C. (2012). MetRxn: a knowledgebase of metabolites and reactions spanning metabolic models and databases. *BMC Bioinformatics*, **13**(1), 6.

Lang, M., Stelzer, M., and Schomburg, D. (2011). BKM-react, an integrated biochemical reaction database. *BMC biochemistry*, **12**(1), 42.

Matos, P. D., Alcantara, R., Dekker, A., Ennis, M., Hastings, J., Haug, K., Spiteri, I., Turner, S., and Steinbeck, C. (2010). Chemical Entities of Biological Interest: an update. *Nucleic Acids Research*, **38**(Database), D249–D254.

Sud, M., Fahy, E., Cotter, D., Brown, A., Dennis, E. A., Glass, C. K., Merrill, A. H., Murphy, R. C., Raetz, C. R. H., Russell, D. W., and Subramaniam, S. (2007). LMSD: LIPID MAPS structure database. *Nucleic Acids Research*, **35**(Database), D527–D532.

Teusink, B., Wiersma, A., Molenaar, D., Francke, C., de Vos, W. M., Siezen, R. J., and Smid, E. J. (2006). Analysis of growth of *Lactobacillus plantarum* WCFS1 on a complex medium using a genome-scale metabolic model. *J Biol Chem*, **281**(52), 40041–8.

The UniProt Consortium (2012). Reorganizing the protein space at the Universal Protein Resource (UniProt). *Nucleic Acids Research*, **40**(D1), D71–D75.

Warr, W. A. (2011). Representation of chemical structures. *WIREs Comput Mol Sci*, **1**(4), 557–579.

Wishart, D. S., Knox, C., Guo, A. C., Eisner, R., Young, N., Gautam, B., Hau, D. D., Psychogios, N., Dong, E., Bouatra, S., Mandal, R., Sinelnikov, I., Xia, J., Jia, L., Cruz, J. A., Lim, E., Sobsey, C. A., Shrivastava, S., Huang, P., Liu, P., Fang, L., Peng, J., Fradette, R., Cheng, D., Tzur, D., Clements, M., Lewis, A., Souza, A. D., Zuniga, A., Dawe, M., Xiong, Y., Clive, D., Greiner, R., Nazyrova, A., Shaykhutdinov, R., Li, L., Vogel, H. J., and Forsythe, I. (2009). HMDB: a knowledgebase for the human metabolome. *Nucleic Acids Research*, **37**(Database), D603–D610.
