# Supplementary material for: Metingear: a development environment for annotating genome-scale metabolic models
Source: Bioinformatics. 2013 Jun 13;29(17):2213–5. doi: 10.1093/bioinformatics/btt342 (PMC3740624; doi:10.1093/bioinformatics/btt342)
Supplement: Supplementary Data [file supp_btt342_tutorial.pdf]

Tutorial: Annotating a Systems Biology Markup Language (SBML) model

This page will guide you through the process of annotating a Systems Biology Markup Language (SBML) model. This tutorial is intended a guide only, further details can be found on the other wiki pages. There is a screencast to accompany this tutorial is available here. The interactive version of this document can be found here.

Before you begin

The model

We will be annotating the a consensus model of *Salmonella typhimurium* LT2 (Theile et al, 2011). To begin, download the SBML from the supplementary material: `1752-0509-5-8-s2.zip` and extract the `STM_v1.0.xml` file to an accessible location on your computer.

Download Metingear

You can download the latest version of Metingear from the home page. Further information on starting the application can be found on the Installation page. **When running the Java Archive version (`.jar`) be sure to provide the Virtual Machine enough memory** Installation/Java Archive.

Resources

With Metingear open, choose the menu item `Edit > Preferences`. Within the `Resources` preferences you can load information from a variety of datasets. Some dataset require the location to be manually defined as their files are either not freely accessibly or are very large. The datasets used in this tutorial can all be automatically downloaded. Although you can load multiple resources at once it is **not** advisable on **large** data sets as this may lead to excessive memory and CPU consumption. If you are having issues when the loading the resources you may not have assigned enough memory to the Java Virtual Machine (see. Installation/Java Archive).

For this tutorial we will need four resources. Click the update icon (down arrow) for `ChEBI Names`, `ChEBI Chemical Structures` **(large)**, `ChEBI Data` and `UniProt Taxonomy` - these are indicated below.

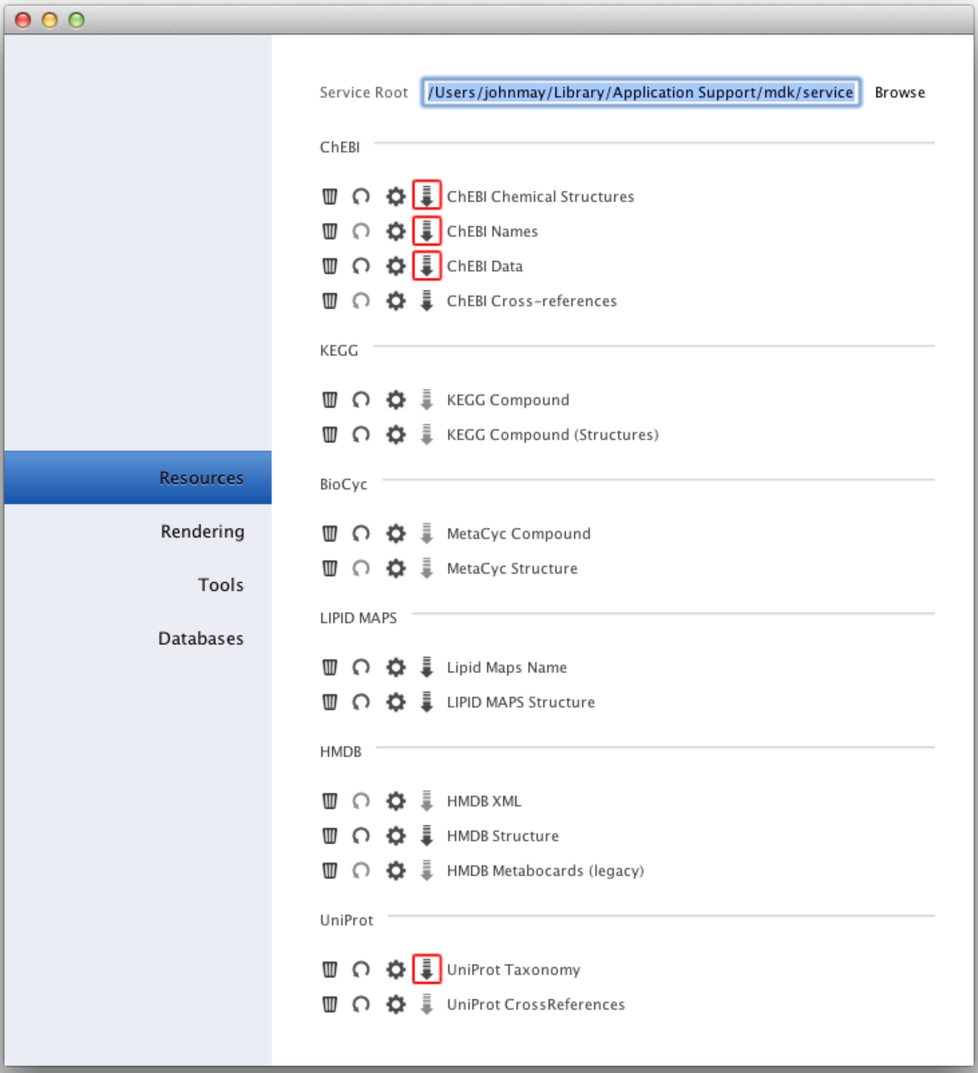

Please refer to the Resources page for a more detailed description of each resource.

Loading the SBML

Create a reconstruction

top|next

Before we open the SBML file we need to create a reconstruction into which the metabolites and reactions can be loaded. Open the Metingear application and select the menu item `File > New Reconstruction`. This will pressent you with a dialog and several text fields. Select the **Organism Code** field and enter `salty` - this is the five character mnemonic from UniProt. If you have successfully loaded the *UniProt Taxonomy* resource entering the code will display a list of possible options below the text field. Select and click the first entry in the drop-down list and the rest of the fields in the dialog will be filled in.

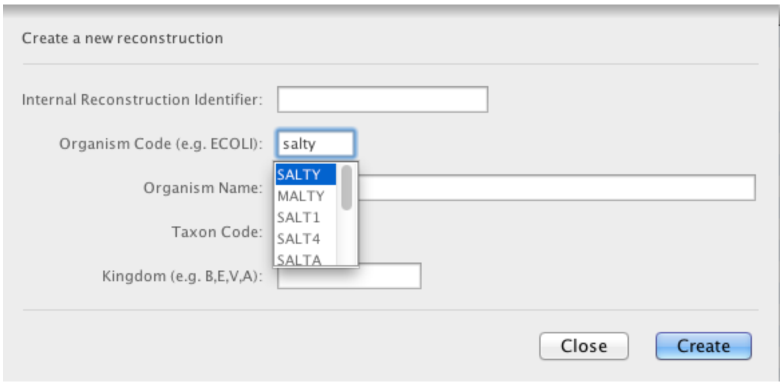

If you have not loaded the *UniProt Taxonomy* resource then you will need to complete each of the fields manually and specify an identifier for your reconstruction. The fields are `Organism Name=Salmonella typhimurium`, `Organism Code=salty`, `Taxon Code=99287`, `Kingdom=BACTERIA`.

Import the SBML file

prev|top|next

Creating a reconstruction will update the sidebar to show there is currently one active reconstruction. With an active reconstruction we can now load our SBML file. Select `File > Import SBML` and use the file chooser to navigate to where you extracted `STM_v1.0.xml` and open it. Please wait while the file is opened - this may take a long time for larger files. Some SBML files may contain compartments that Metingear does not yet recognise in this case a popup will request you select an appropriate compartment (see Import).

Once the SMBL has been imported the side bar should update with labels beside `Metabolites` and `Reactions`. These labels list the total count of that entity type.

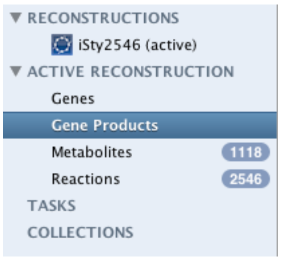

To view the metabolites and reactions you must navigate to either the `Metabolites` or `Reactions` view. It is possible to navigate to the view by selecting `Metabolites` or `Reactions` in the side bar. On the toolbar at the top there is a button group which list the view you are currently on. To navigate to another view click one of buttons which is not depressed.

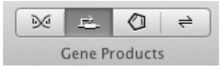

Saving the active reconstruction

prev|top|next

Before we continue it is a good idea to save the reconstruction by selecting `File > Save`. This will save the reconstruction as binary, the default location to save is `<home>/<recon.id>.mr`. In my case the reconstruction id was `iSty2546` and so the reconstruction was saved to `/Users/johnmay/iSty2546.mr` (see also Saving Internally).

Â Extracting Annotations

Selecting metabolites

prev|top|next

With the model loaded select the `Metabolites` view and click on the `Accession` column. This will sort the entries by accession. Click on the accession column until the rows are sorted, the first entries should be; `M_10fthf_c`, `M_12dgr120_p`, `M_12dgr140_p`, etc.

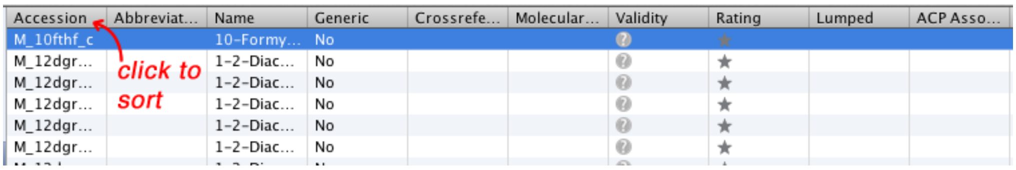

Select the first entry in the table `M_10fthf_c` - *10-Formyltetrahydrofolate*. Notice how the inspector ad the bottom changes. On the right of the inspector there are several *Notes* defined for this entity.

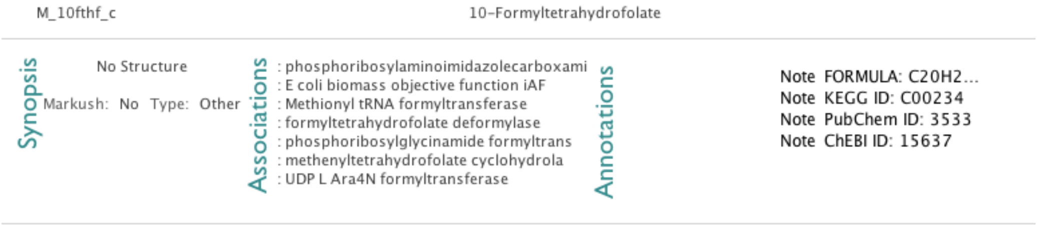

These annotations correspond to the `<notes>` element of the imported SBML.

```
<species id="M_10fthf_c" name="10-Formyltetrahydrofolate" compartment="c" charge="-2">
  <notes>
    <html xmlns="http://www.w3.org/1999/xhtml">
      <p>FORMULA: C20H21N7O7</p>
      <p>KEGG ID: C00234</p>
      <p>PubChem ID: 3533</p>
      <p>ChEBI ID: 15637</p>
    </html>
  </notes>
</species>
```

There are currently two tools in Metingear which allow us to extract information from the *Notes*. Extracting the information gives the data context and definition which is required to unambiguously identify what a metabolite is.

Extracting cross-references

prev|top|next

Whilst viewing the `Metabolites` select the first *5* entries (`M_10fthf_c` - `M_12dgr160_p`).

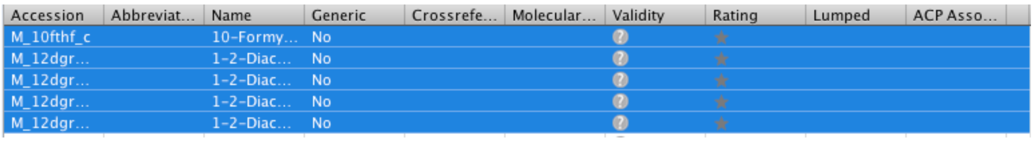

With the entries selected, click the menu item `Tools > Annotation > Extract cross-references from notes`.

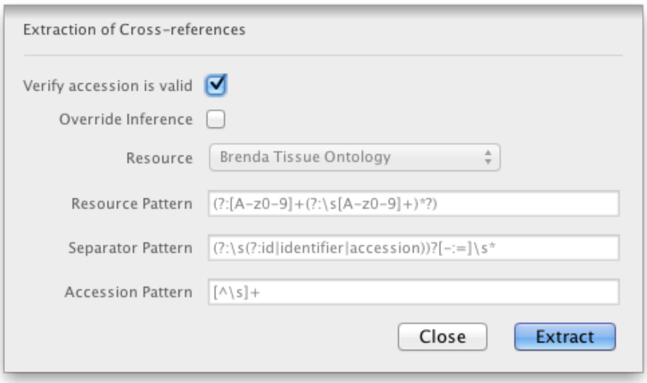

Without changing any options in the dialog, click `Extract`. This will update the list of metabolites which will now have `ChEBI` cross-references listed in the `Cross-references` column and in the inspector.

Accession	Abbreviat...	Name	Generic	Cross-reference	Molecular...	Validity	Rating	Lumped
M_10fthf_c		10-Formy...	No	CHEBI:15637				
M_12dgr...		1-2-Diac...	No	CHEBI:17815				
M_12dgr...		1-2-Diac...	No	CHEBI:17815				
M_12dgr...		1-2-Diac...	No	CHEBI:17815				

Selecting the first entry we can also see the annotation table has updated. Clicking the value (`CHEBI:15637`) for the `ChEBI Cross-reference` will open up the entry in your default browser.

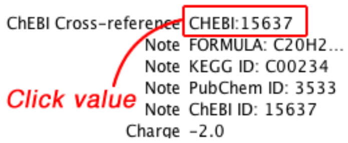

With the `ChEBI` references loaded we will now undo this action. Select `Edit > Undo` to remove the added cross-references. The metabolites should update and the first 5 entries will no longer have any cross-references listed.

Accession	Abbreviat...	Name	Generic	Crossrefe...	Molecular...	Validity	Rating	Lumped	ACP Asso...
M_10fthf_c		10-Formy...	No						
M_12dgr...		1-2-Diac...	No						
M_12dgr...		1-2-Diac...	No						
M_12dgr...		1-2-Diac...	No						
M_12dgr...		1-2-Diac...	No						

You may have noticed that the `PubChem ID` and `KEGG ID` references were not extracted by the tool. This is because the names `PubChem` and `KEGG` are too general to infer which resource the identifier was from. In the case of `PubChem` this could be from `PubChem-Compound` (`CIDs`) or from `PubChem-Substance` (`SIDs`). Generally `PubChem-Compound` is more common as `PubChem-Subtance` contains redundant entries. Unfortunately there is no difference in the identifier format between these two databases. The `PubChem ID: 3533` from the first reference could be either a `CID` or an `SID`. As is shown below the entries for `CID 3533` and `SID 3533` are not the same.

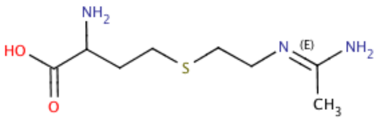

CID 3533

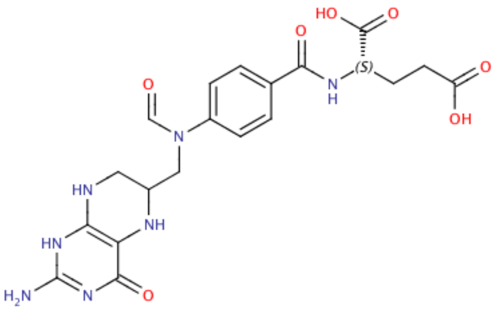

SID 3533

This metabolite was actually referencing the `SID` but cannot infer this from the identifier alone.

In the case of `KEGG`, metabolic models normally refer to `KEGG LIGAND` which is actually a composition of several databases including: `KEGG COMPOUND`, `KEGG GLYCAN`, `KEGG DRUG` and `KEGG REACTION`. In this case it looks like all the identifiers are for `KEGG COMPOUND` in which case we must tell the extraction dialog that `KEGG` is an alias for `KEGG COMPOUND`. If one of the `KEGG ID` identifiers is not from `KEGG COMPOUND` then it will only be included if `Verify accession is valid` is unchecked.

Select the first 5 entries again and click the menu item `Tools > Annotation > Extract cross-references from notes`. This time we are going to check the `Override Inference` box and select `KEGG COMPOUND` from the list of resources.

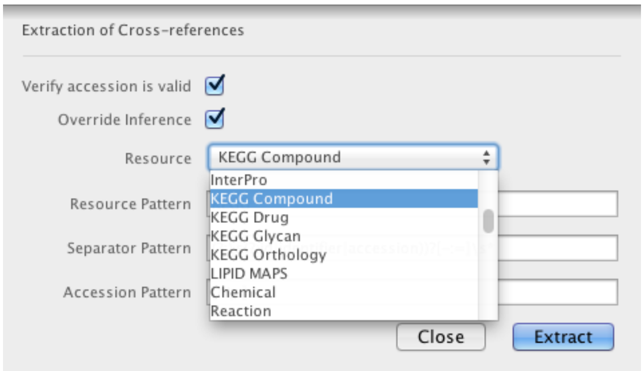

The `Resource Pattern` will update and now contain `KEGG COMPOUND`. As we are specifying `KEGG` as an alais for `KEGG COMPOUND` we must remove the `COMPOUND` suffix.

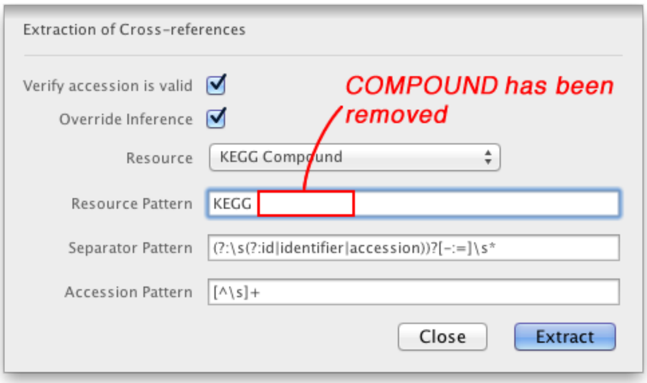

With the suffix removed we can run the tool again by clicking `Extract`. This time the cross-references `C000234` and `C000641` are correctly extracted and assigned.

Accession	Abbreviat...	Name	Generic	Crossreference
M_10fthf_c		10-Formy...	No	C00234
M_12dgr120_p		1-2-Diac...	No	C00641
M_12dgr140_p		1-2-Diac...	No	C00641
M_12dgr141_p		1-2-Diac...	No	C00641
M_12dgr160_p		1-2-Diac...	No	C00641

Extracting textual annotations

prev|top|next

In addition to the the cross-references we also have *Notes* which contain the formula of the metabolites. We can extract the formula and other *textual* annotations in a similar manner to the cross-references. Select the first 5 metabolites again and click the menu item `Tool > Annotations > Extract textual annotations from notes`.

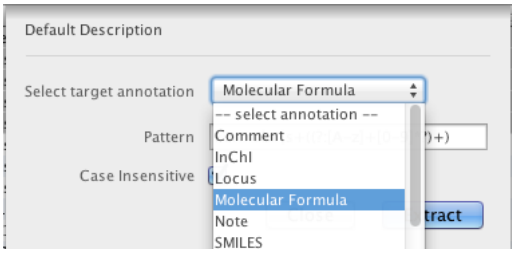

Select `Molecular Formula` from the combo box, this will set the `Pattern` required to extract the formula. Some *Notes* may require a different `Pattern` but for this model the default option is okay. Click `Extract` and the formula for the first 5 entries will be added. Please refer to the Tools page for more information on extracting cross-references and text annotations.

Accession	Abbreviat...	Name	Generic	Crossreference	Molecular...
M_10fthf_c		10-Formyltetrahydrofolate	No	C00234	C20H21N...
M_12dgr120_p		1-2-Diacyl-sn-glycerol-didodecanoyl-n-C120	No	C00641	C27H52O5
M_12dgr140_p		1-2-Diacyl-sn-glycerol-ditetradecanoyl-n-C140	No	C00641	C31H60O5
M_12dgr141_p		1-2-Diacyl-sn-glycerol-ditetradec-7-enoyl-n-C141	No	C00641	C31H56O5
M_12dgr160_p		1-2-Diacyl-sn-glycerol-dihexadecanoyl-n-C160	No	C00641	C35H68O5

Assigning Annotations

In addition to extracting annotations from notes it is also possible to add annotations manually or to infer an annotation based on a metabolite name. This section will guide you through these steps as well as how to attach a chemical structure when the no cross-reference is adequate.

Manual cross-reference

prev|top|next

We are going to search for and add a cross-reference to `1-4-alpha-D-glucan`. To search for this metabolite we will use the search box at the top right of the toolbar. Enter `"1-4-alpha-D-glucan"` in the **search box** making sure to include the **quotation marks**. The quotations are required as this name contains the subtract (`-`) character which can be used to narrow the search and exclude terms you do not want to appear in the results.

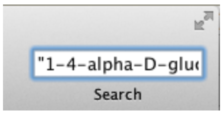

Entering the name will update the active view to show the entities which match your query.

Rank	Accession	Abbreviat...	Name	Type
1	M_14glucan_p		1-4-alph...	Metabolit...
2	R_EX_14glucan_e		1 4 alpha...	Metabolic...
3	R_14GLUCANabcpp		1 4 alpha...	Metabolic...
4	R_14GLUCANtexi		1 4 alpha...	Metabolic...
5	R_AAMYL		alpha amy...	Metabolic...
6	R_AAMYLpp		alpha amy...	Metabolic...

In this case the first entry is the one we wanted.

Rank	Accession	Abbreviat...	Name	Type
1	M_14glucan_p		1-4-alph...	MetaboliteImpl
2	R_EX_14glucan_e		1 4 alpha...	MetabolicReactionImpl
3	R_14GLUCANabcpp		1 4 alpha...	MetabolicReactionImpl
4	R_14GLUCANtexi		1 4 alpha...	MetabolicReactionImpl
5	R_AAMYL		alpha amy...	MetabolicReactionImpl
6	R_AAMYLpp		alpha amy...	MetabolicReactionImpl

Double click on this entry and you will be taken back to the `Metabolites` view with `1-4-alpha-D-glucan` as the selected entry.

Accession	Abbreviat...	Name	Generic	Crossrefe...	Molecular...	Validity	Rating
M_12dgr...		1-2-Diac...	No				
M_12dgr...		1-2-Diac...	No				
M_12dgr...		1-2-Diac...	No				
M_12dgr...		1-2-Diac...	No				
M_12dgr...		1-2-Diac...	No				
M_12dgr...		Membran...	No				
M_12ppd...		R-Propan...	No				
M_12ppd...		S-Propan...	No				
M_13dpg_c		3-Phosph...	No				
M_14gluc...		1-4-alph...	No				
M_15dap_c		1-5-Diam...	No				
M_1agpe...		1-Acyl-s...	No				
M_1agpe...		1-Acyl-s...	No				
M_1agpe...		1-Acyl-s...	No				
M_1agpe...		1-Acyl-s...	No				
M_1agpe...		1-Acyl-s...	No				
M_1agpe...		1-Acyl-s...	No				
M_1agpe...		1-Acyl-s...	No				
M_1agpg...		1-Acyl-s...	No				
M_1agpg...		1-Acyl-s...	No				

In the inspector we can see this entry has a *Note* for the `KEGG COMPOUND` identifier but the `ChEBI` and `PubChem-Compound` references are empty (`NA`). As of July 2012 (after this model was published) `ChEBI` now has `(1â†’4)-Î±-D-glucan (CHEBI:15444)` which we can add as a cross-reference. To manually add this cross-reference double click on the the cross-reference column. This will open a callout dialog with a text-field.

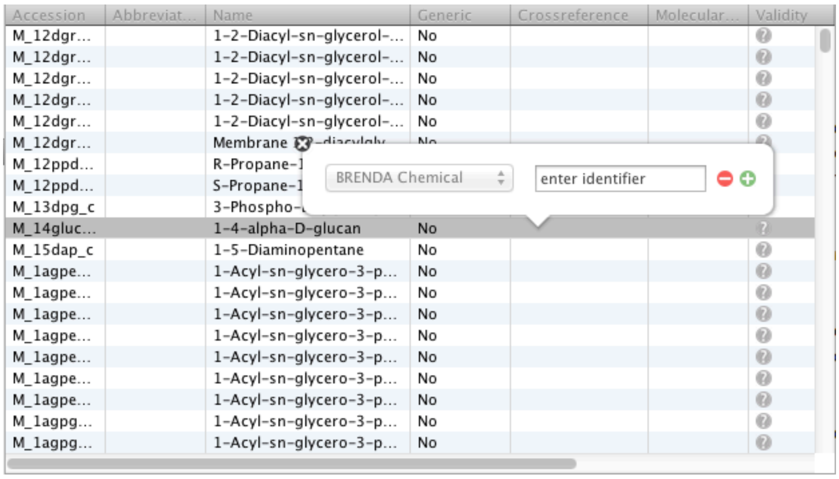

Select the text field in the dialog and enter `CHEBI:15444` this will automatically update the resource selection on the left to `ChEBI`.

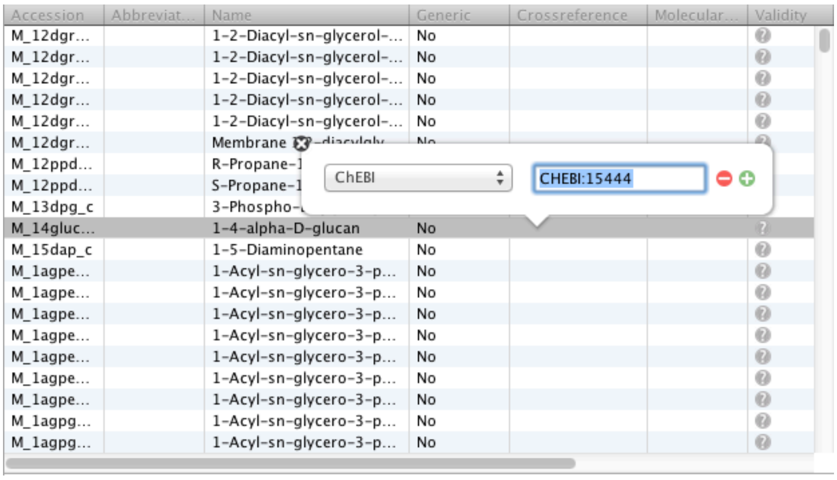

We can also add additional cross-references such as the KEGG COMPOUND identifier listed which was listed in the *Notes*. To add another identifier, click the green plus to the right of the text-field. This will add another row to the dialog.

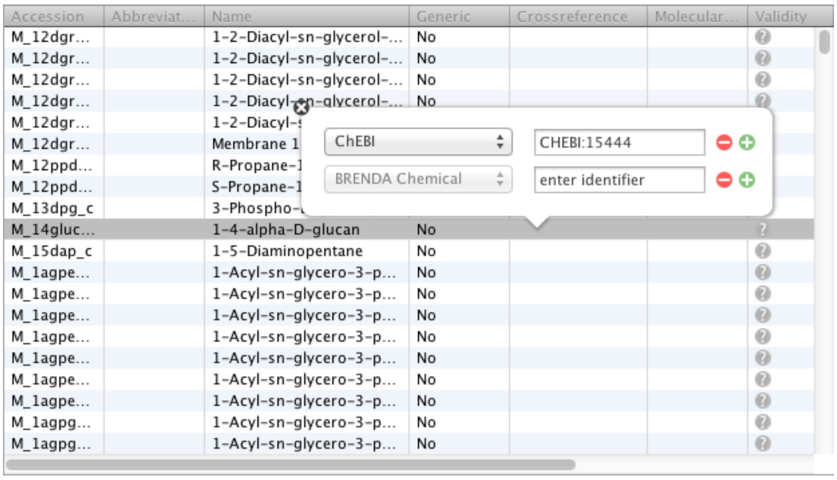

Enter the KEGG COMPOUND identifier `C00912` into this second field.

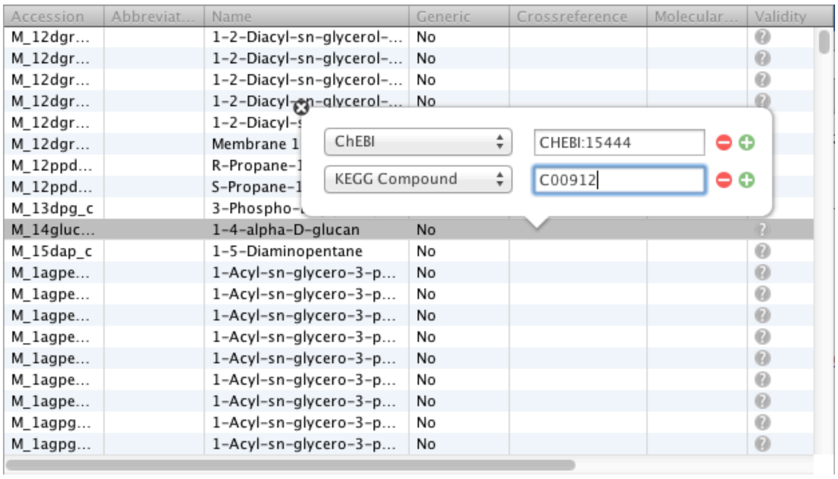

Accession	Abbreviat…	Name	Generic	Crossreference	Molecular…	Validity
M_12dgr…		1-2-Diacyl-sn-glycerol-…	No			
M_12dgr…		1-2-Diacyl-sn-glycerol-…	No			
M_12dgr…		1-2-Diacyl-sn-glycerol-…	No			
M_12dgr…		1-2-Diacyl-sn-glycerol-…	No			
M_12dgr…		1-2-Diacyl-[illegible]				
M_12dgr…		Membrane 1[illegible]				
M_12ppd…		R-Propane-1[illegible]				
M_12ppd…		S-Propane-1[illegible]				
M_13dpg_c		3-Phospho-[illegible]				
M_14gluc…		1-4-alpha-D-glucan	No			
M_15dap_c		1-5-Diaminopentane	No			
M_1agpe…		1-Acyl-sn-glycero-3-p…	No			
M_1agpe…		1-Acyl-sn-glycero-3-p…	No			
M_1agpe…		1-Acyl-sn-glycero-3-p…	No			
M_1agpe…		1-Acyl-sn-glycero-3-p…	No			
M_1agpe…		1-Acyl-sn-glycero-3-p…	No			
M_1agpe…		1-Acyl-sn-glycero-3-p…	No			
M_1agpe…		1-Acyl-sn-glycero-3-p…	No			
M_1agpg…		1-Acyl-sn-glycero-3-p…	No			
M_1agpg…		1-Acyl-sn-glycero-3-p…	No			

Again the resource selection has correctly updated to `KEGG Compound`. Closing the dialog using the cross in the top left will add the cross-references to the entity.

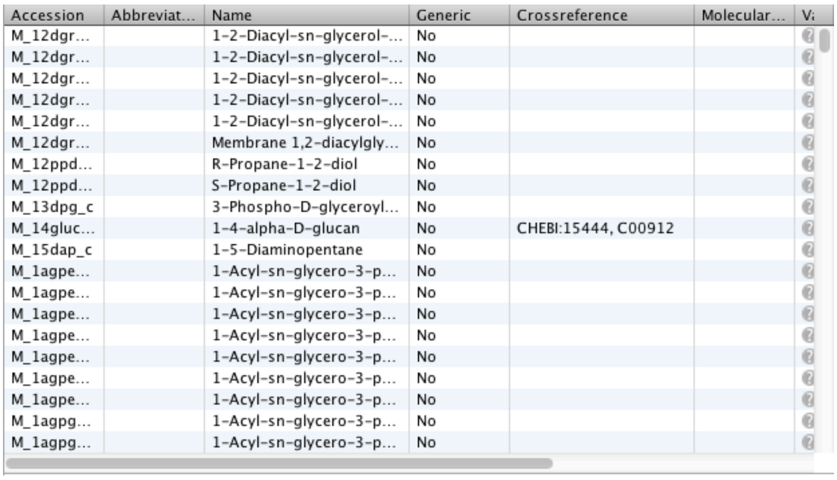

Accession	Abbreviat…	Name	Generic	Crossreference	Molecular…	V:
M_12dgr…		1-2-Diacyl-sn-glycerol-…	No			
M_12dgr…		1-2-Diacyl-sn-glycerol-…	No			
M_12dgr…		1-2-Diacyl-sn-glycerol-…	No			
M_12dgr…		1-2-Diacyl-sn-glycerol-…	No			
M_12dgr…		1-2-Diacyl-sn-glycerol-…	No			
M_12dgr…		Membrane 1,2-diacylgly…	No			
M_12ppd…		R-Propane-1-2-diol	No			
M_12ppd…		S-Propane-1-2-diol	No			
M_13dpg_c		3-Phospho-D-glyceroyl…	No			
M_14gluc…		1-4-alpha-D-glucan	No	CHEBI:15444, C00912		
M_15dap_c		1-5-Diaminopentane	No			
M_1agpe…		1-Acyl-sn-glycero-3-p…	No			
M_1agpe…		1-Acyl-sn-glycero-3-p…	No			
M_1agpe…		1-Acyl-sn-glycero-3-p…	No			
M_1agpe…		1-Acyl-sn-glycero-3-p…	No			
M_1agpe…		1-Acyl-sn-glycero-3-p…	No			
M_1agpe…		1-Acyl-sn-glycero-3-p…	No			
M_1agpe…		1-Acyl-sn-glycero-3-p…	No			
M_1agpg…		1-Acyl-sn-glycero-3-p…	No			
M_1agpg…		1-Acyl-sn-glycero-3-p…	No			

Automatic cross-reference

prev|top|next

Although it is possible to manually add each cross-reference it is a lot faster to do this automatically. We can use the name of a metabolite to find an appropriate cross-reference from our loaded resources. Select the entries `R-Propane-1-2-diol` and `S-Propane-1-2-diol` - these entries should be just above `1-4-alpha-D-glucan` when sorted by accession (see previous paragraph).

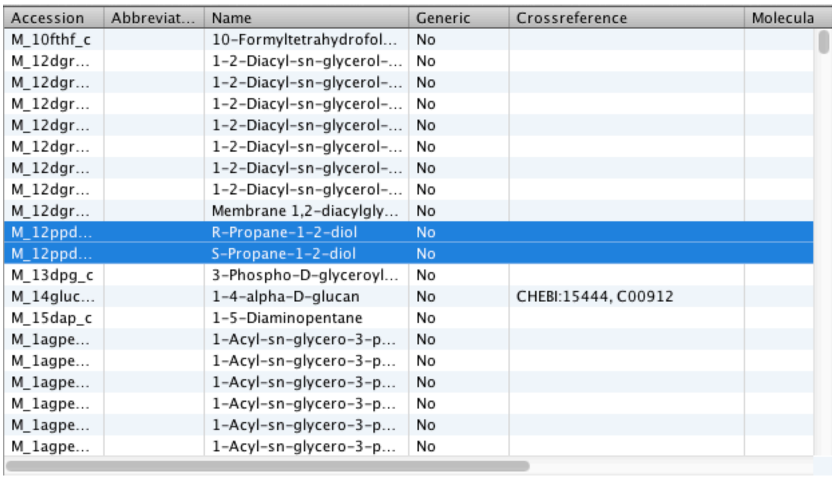

Accession	Abbreviat…	Name	Generic	Crossreference	Molecula
M_10fthf_c		10-Formyltetrahydrofol…	No		
M_12dgr…		1-2-Diacyl-sn-glycerol-…	No		
M_12dgr…		1-2-Diacyl-sn-glycerol-…	No		
M_12dgr…		1-2-Diacyl-sn-glycerol-…	No		
M_12dgr…		1-2-Diacyl-sn-glycerol-…	No		
M_12dgr…		1-2-Diacyl-sn-glycerol-…	No		
M_12dgr…		1-2-Diacyl-sn-glycerol-…	No		
M_12dgr…		1-2-Diacyl-sn-glycerol-…	No		
M_12dgr…		Membrane 1,2-diacylgly…	No		
M_12ppd…		R-Propane-1-2-diol	No		
M_12ppd…		S-Propane-1-2-diol	No		
M_13dpg_c		3-Phospho-D-glyceroyl…	No		
M_14gluc…		1-4-alpha-D-glucan	No	CHEBI:15444, C00912	
M_15dap_c		1-5-Diaminopentane	No		
M_1agpe…		1-Acyl-sn-glycero-3-p…	No		
M_1agpe…		1-Acyl-sn-glycero-3-p…	No		
M_1agpe…		1-Acyl-sn-glycero-3-p…	No		
M_1agpe…		1-Acyl-sn-glycero-3-p…	No		
M_1agpe…		1-Acyl-sn-glycero-3-p…	No		
M_1agpe…		1-Acyl-sn-glycero-3-p…	No		

Click the menu item `Tools > Annotation > Automatic Cross-reference` this will open up a dialog with several options. In the

opened dialog select the checkbox `Approximate match`.

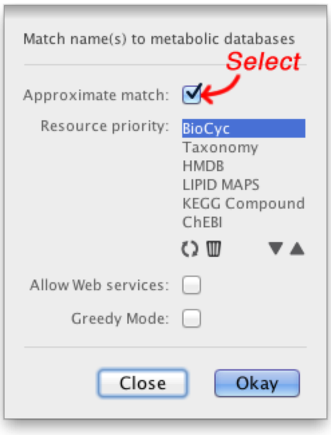

If you have more then one resource available drag ChEBI to the top of the `Resource priority`.

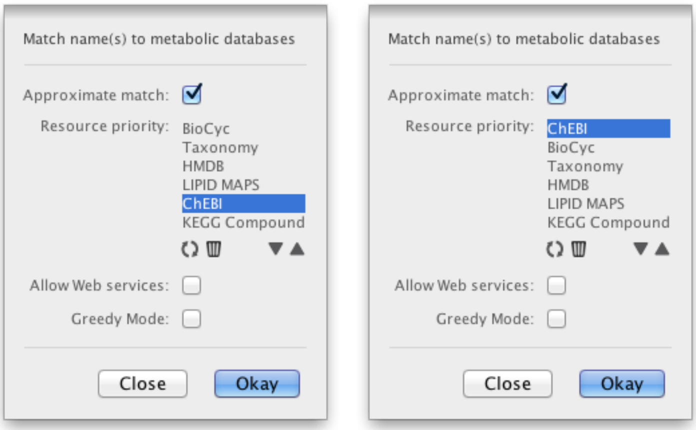

Selecting `Okay` will perform a search on the name of each metabolite and attempt to assign one or more cross-references to the selected metabolites. The search succeeds and adds `CHEBI:28792` and `CHEBI:29002` as Cross-references on these metabolites.

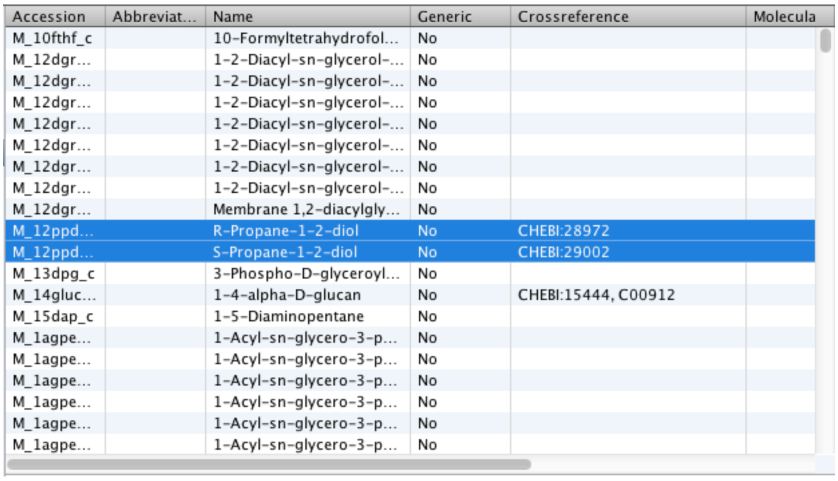

Accession	Abbreviat...	Name	Generic	Crossreference	Molecula
M_10fthf_c		10-Formyltetrahydrofol...	No		
M_12dgr...		1-2-Diacyl-sn-glycerol-...	No		
M_12dgr...		1-2-Diacyl-sn-glycerol-...	No		
M_12dgr...		1-2-Diacyl-sn-glycerol-...	No		
M_12dgr...		1-2-Diacyl-sn-glycerol-...	No		
M_12dgr...		1-2-Diacyl-sn-glycerol-...	No		
M_12dgr...		1-2-Diacyl-sn-glycerol-...	No		
M_12dgr...		1-2-Diacyl-sn-glycerol-...	No		
M_12dgr...		Membrane 1,2-diacylgly...	No		
M_12ppd...		R-Propane-1-2-diol	No	CHEBI:28972	
M_12ppd...		S-Propane-1-2-diol	No	CHEBI:29002	
M_13dpg_c		3-Phospho-D-glyceroyl...	No		
M_14gluc...		1-4-alpha-D-glucan	No	CHEBI:15444, C00912	
M_15dap_c		1-5-Diaminopentane	No		
M_1agpe...		1-Acyl-sn-glycero-3-p...	No		
M_1agpe...		1-Acyl-sn-glycero-3-p...	No		
M_1agpe...		1-Acyl-sn-glycero-3-p...	No		
M_1agpe...		1-Acyl-sn-glycero-3-p...	No		
M_1agpe...		1-Acyl-sn-glycero-3-p...	No		
M_1agpe...		1-Acyl-sn-glycero-3-p...	No		

Please refer to Tools/Automatic Cross-reference for more details on using this dialog.

Curated cross-reference

In addition to automatically assigning a cross-reference it can be beneficial to choose the most appropriate. Select the metabolite `3-Phospho-D-glyceroyl-phosphate` this should be just below the two entries from the previous paragraph (if ordered by accession). With the entity select extract the molecular formula from the Note (see [Extract Textual Annotation](#)).

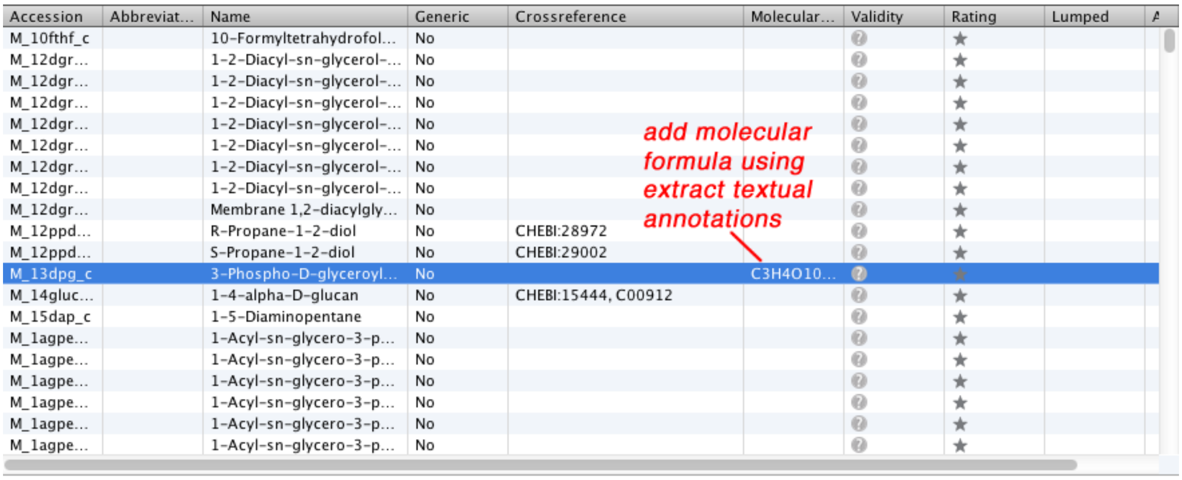

This entry already has several cross-references in the notes, including one for `ChEBI`. The existing `ChEBI` cross-reference (`CHEBI:16001`) is referring to the same structure but at a different protonation state. We will try to find a cross-reference which matches this metabolites protonation state. With the entity selected click the menu item `Tool > Annotations > Curate Metabolite`. This will open a dialog with several sections which are hidden by default. Clicking any arrow on the left will expand that section.

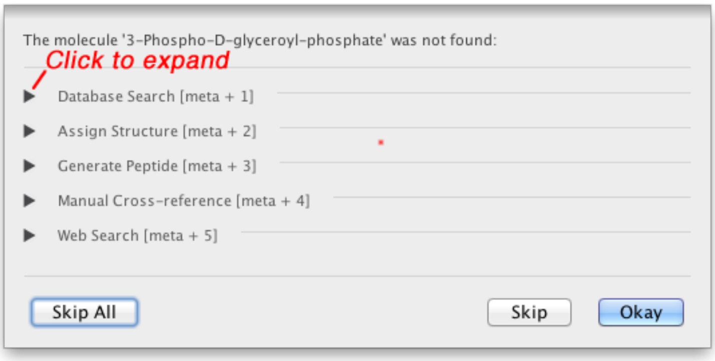

Click the arrow next to the first section *Database Search* to expand that section.

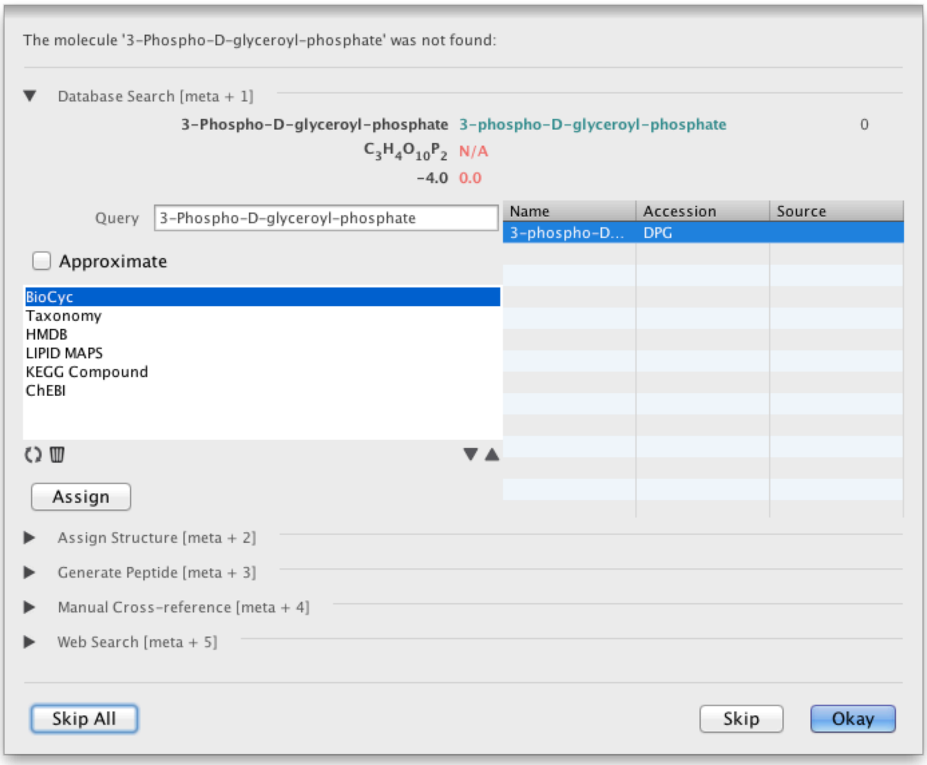

From the list of resources on the left if multiple are available drag `ChEBI` to the top of the list.

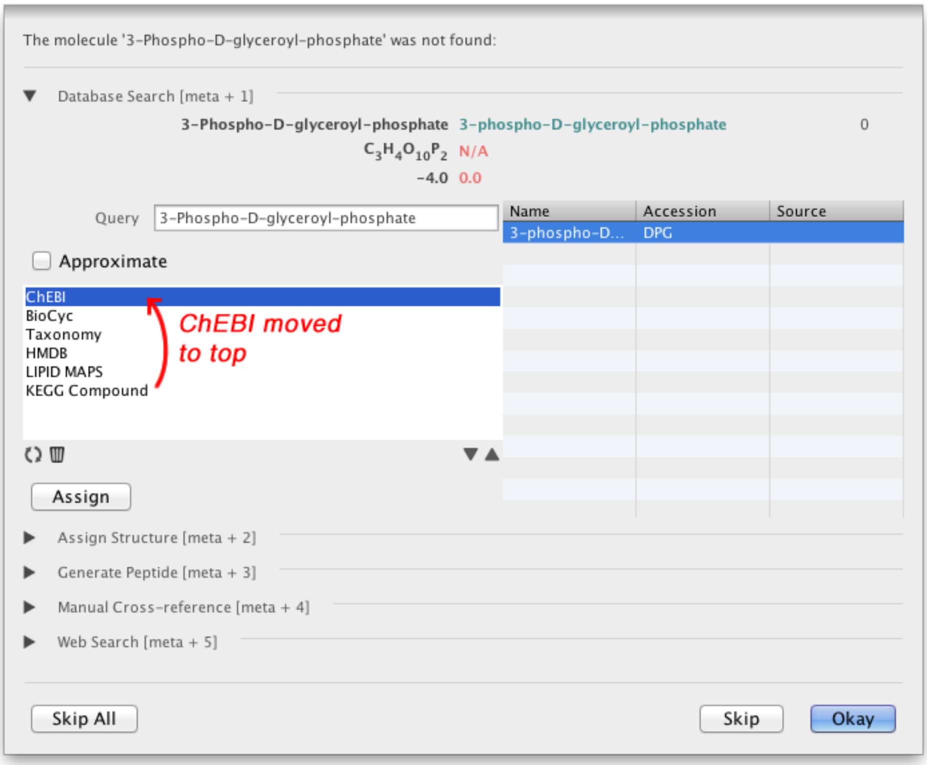

Selecting the `Approximate` box - this will display several results in the table to the right.

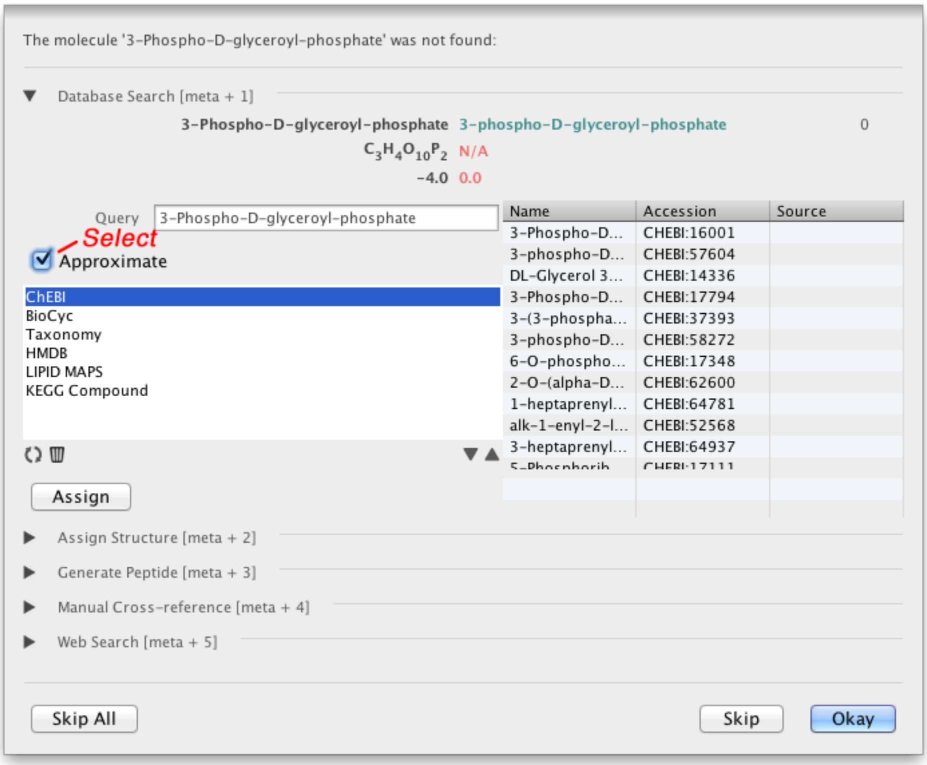

By selecting the first row we can inspect the quality of this reference using the *match indicator* at the top of the section.

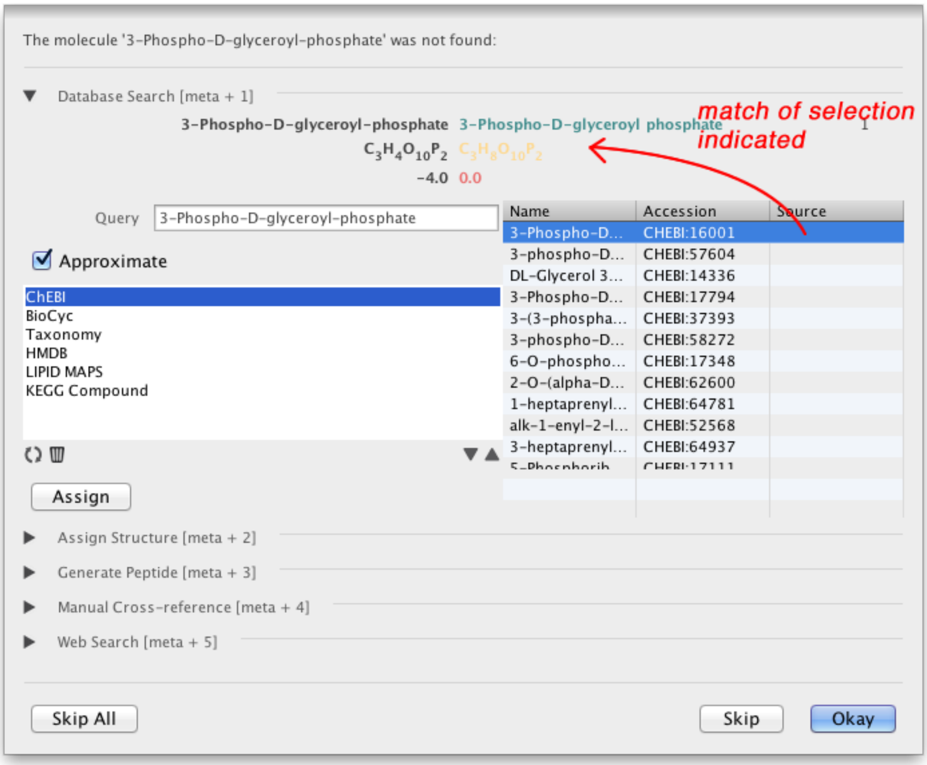

This first entry (`CHEBI:16001`) is the same as the one in the notes. As you can see the match indicates the formula and charge do not match. An orange formula indicates it would be correct if the charge matched. Selecting the second row will change the *match indicator* to show all fields have a successful match.

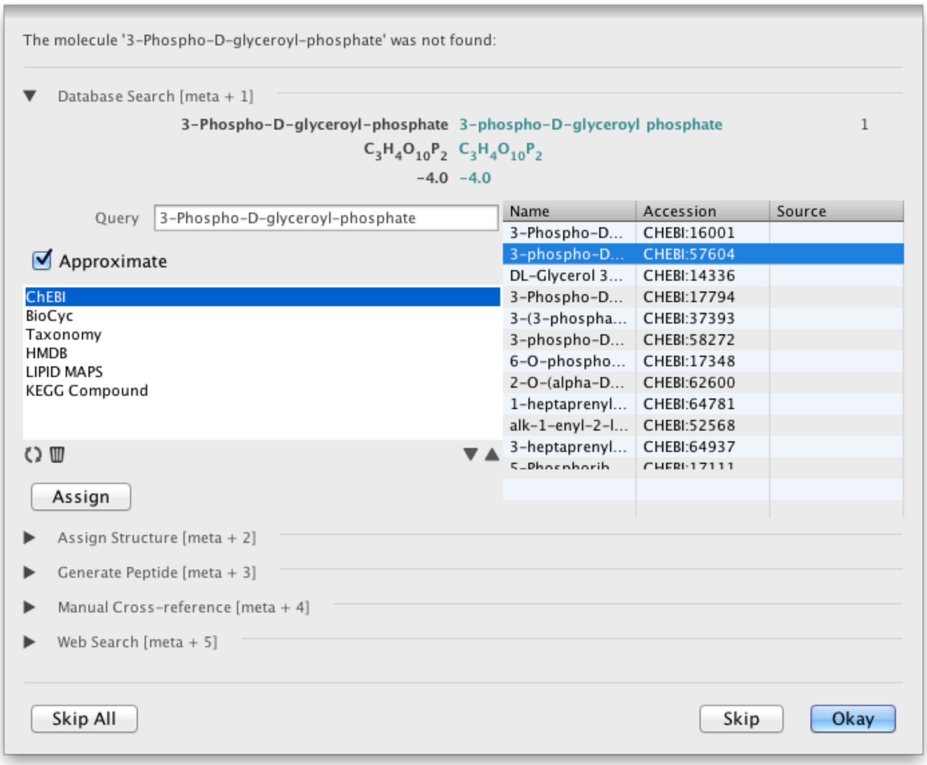

With the second row selected click the `Assign` button at the lower left of the dialog to set this cross-reference on the metabolite.

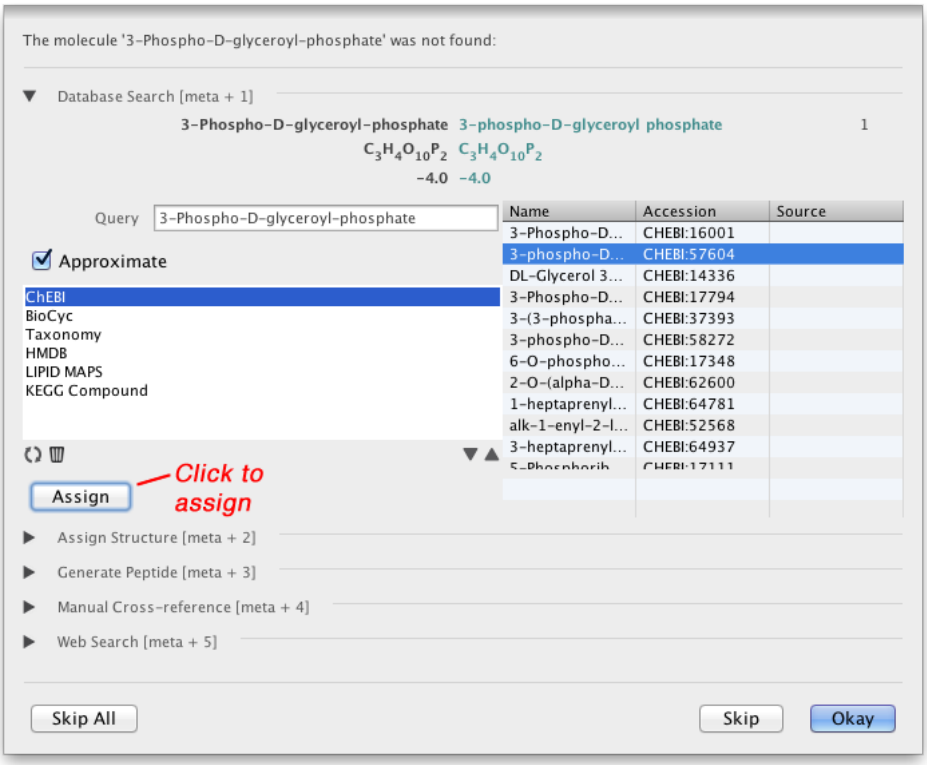

Select `Okay` at the bottom right to close the dialog, the entry is now updated with the correct cross-reference.

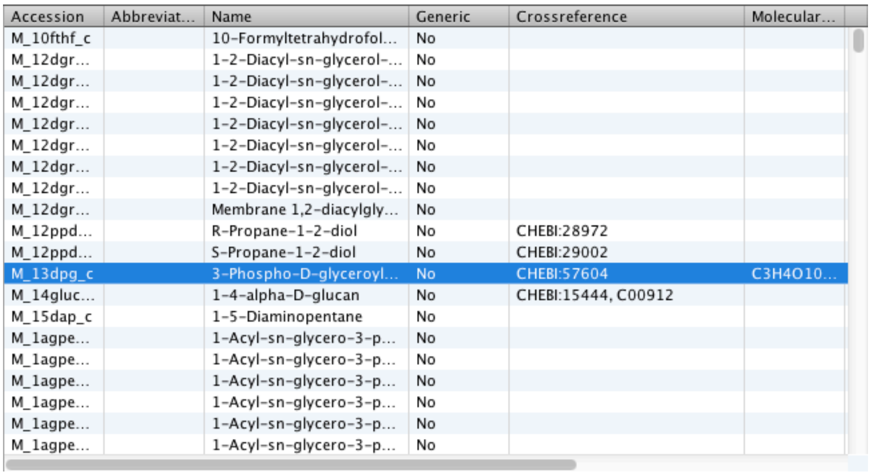

Accession	Abbreviat...	Name	Generic	Crossreference	Molecular...
M_10fthf_c		10-Formyltetrahydrofol...	No		
M_12dgr...		1-2-Diacyl-sn-glycerol-...	No		
M_12dgr...		1-2-Diacyl-sn-glycerol-...	No		
M_12dgr...		1-2-Diacyl-sn-glycerol-...	No		
M_12dgr...		1-2-Diacyl-sn-glycerol-...	No		
M_12dgr...		1-2-Diacyl-sn-glycerol-...	No		
M_12dgr...		1-2-Diacyl-sn-glycerol-...	No		
M_12dgr...		1-2-Diacyl-sn-glycerol-...	No		
M_12dgr...		Membrane 1,2-diacylgly...	No		
M_12ppd...		R-Propane-1-2-diol	No	CHEBI:28972	
M_12ppd...		S-Propane-1-2-diol	No	CHEBI:29002	
M_13dpg_c		3-Phospho-D-glyceroyl...	No	CHEBI:57604	C3H4O10...
M_14gluc...		1-4-alpha-D-glucan	No	CHEBI:15444, C00912	
M_15dap_c		1-5-Diaminopentane	No		
M_1agpe...		1-Acyl-sn-glycero-3-p...	No		
M_1agpe...		1-Acyl-sn-glycero-3-p...	No		
M_1agpe...		1-Acyl-sn-glycero-3-p...	No		
M_1agpe...		1-Acyl-sn-glycero-3-p...	No		
M_1agpe...		1-Acyl-sn-glycero-3-p...	No		
M_1agpe...		1-Acyl-sn-glycero-3-p...	No		

Please refer to Tools/Curate Metabolite for more details on this database search dialog.

Transfer chemical structure

prev|top|next

Now we have some cross-references assigned we can use these to attach a chemical structure. Chemical structure is *transfer* from one or more cross-reference. As the cross-references are linked to a resource we know where to go to find the structure. With the metabolites sorted by accession, select all the rows we have previously edited.

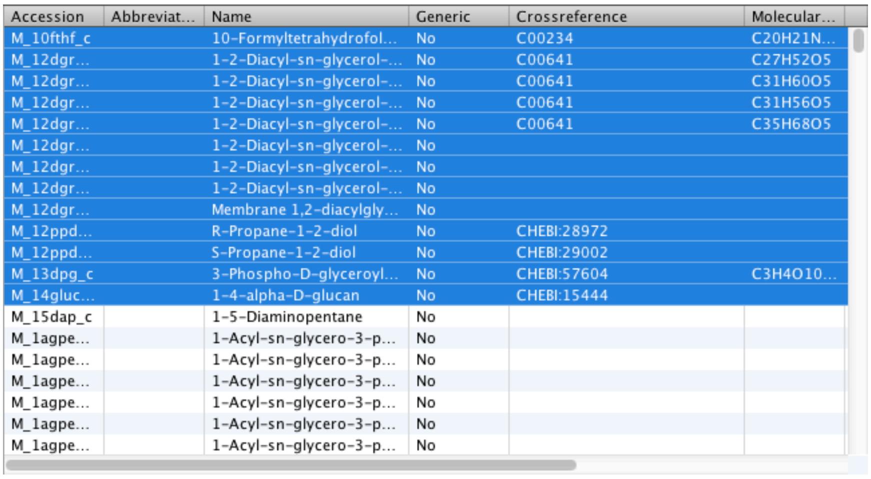

Accession	Abbreviat...	Name	Generic	Crossreference	Molecular...
M_10fthf_c		10-Formyltetrahydrofol...	No	C00234	C20H21N...
M_12dgr...		1-2-Diacyl-sn-glycerol-...	No	C00641	C27H52O5
M_12dgr...		1-2-Diacyl-sn-glycerol-...	No	C00641	C31H60O5
M_12dgr...		1-2-Diacyl-sn-glycerol-...	No	C00641	C31H56O5
M_12dgr...		1-2-Diacyl-sn-glycerol-...	No	C00641	C35H68O5
M_12dgr...		1-2-Diacyl-sn-glycerol-...	No		
M_12dgr...		1-2-Diacyl-sn-glycerol-...	No		
M_12dgr...		1-2-Diacyl-sn-glycerol-...	No		
M_12dgr...		Membrane 1,2-diacylgly...	No		
M_12ppd...		R-Propane-1-2-diol	No	CHEBI:28972	
M_12ppd...		S-Propane-1-2-diol	No	CHEBI:29002	
M_13dpg_c		3-Phospho-D-glyceroyl...	No	CHEBI:57604	C3H4O10...
M_14gluc...		1-4-alpha-D-glucan	No	CHEBI:15444	
M_15dap_c		1-5-Diaminopentane	No		
M_1agpe...		1-Acyl-sn-glycero-3-p...	No		
M_1agpe...		1-Acyl-sn-glycero-3-p...	No		
M_1agpe...		1-Acyl-sn-glycero-3-p...	No		
M_1agpe...		1-Acyl-sn-glycero-3-p...	No		
M_1agpe...		1-Acyl-sn-glycero-3-p...	No		
M_1agpe...		1-Acyl-sn-glycero-3-p...	No		

Choose the menu item `Tools > Annotation > Transfer Chemical Structure`. This will open the following dialog with several options. Please refer to Tools for a detail explanation of the available options.

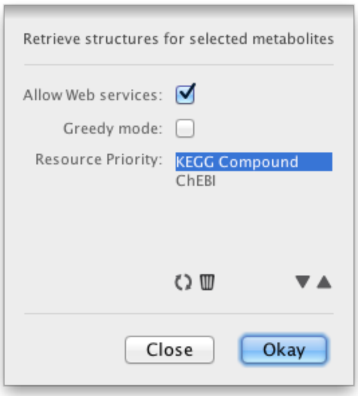

Selecting `Allow Web services` will allow you to fetch structures from `KEGG Compound`. The resources will be processed in the order they

appear. To follow this tutorial `ChEBI` should be moved to the top. With the options specified, click `Okay`. The `Metabolites` table will update and the entries now have chemical structures attached.

Accession	Abbreviat...	Name	Generic	Crossreference	Molecular...	Validity
M_10fthf_c		10-Formyltetrahydrofol...	No	C00234	C20H21N...	!
M_12dgr...		1-2-Diacyl-sn-glycerol-...	No	C00641	C27H52O5	✖
M_12dgr...		1-2-Diacyl-sn-glycerol-...	No	C00641	C31H60O5	✖
M_12dgr...		1-2-Diacyl-sn-glycerol-...	No	C00641	C31H56O5	✖
M_12dgr...		1-2-Diacyl-sn-glycerol-...	No	C00641	C35H68O5	✖
M_12dgr...		1-2-Diacyl-sn-glycerol-...	No			?
M_12dgr...		1-2-Diacyl-sn-glycerol-...	No			?
M_12dgr...		1-2-Diacyl-sn-glycerol-...	No			?
M_12dgr...		Membrane 1,2-diacylgly...	No			?
M_12ppd...		R-Propane-1-2-diol	No	CHEBI:28972		✖
M_12ppd...		S-Propane-1-2-diol	No	CHEBI:29002		✖
M_13dpg_c		3-Phospho-D-glyceroyl...	No	CHEBI:57604	C3H4O10...	✔
M_14gluc...		1-4-alpha-D-glucan	No	CHEBI:15444		✖
M_15dap_c		1-5-Diaminopentane	No			?
M_1agpe...		1-Acyl-sn-glycero-3-p...	No			?
M_1agpe...		1-Acyl-sn-glycero-3-p...	No			?
M_1agpe...		1-Acyl-sn-glycero-3-p...	No			?
M_1agpe...		1-Acyl-sn-glycero-3-p...	No			?
M_1agpe...		1-Acyl-sn-glycero-3-p...	No			?
M_1agpe...		1-Acyl-sn-glycero-3-p...	No			?

To the right of each row in the table (you may need to scroll) an icon indicates whether a structure is likely to be correct (`Validity`). The `Validity` is calculated given an annotated *Charge* and *Molecular Formula*. The two structures for `R-Propane-1-2-diol` and `S-Propane-1-2-diol` are indicated in red because the formula has not been attached. As before, we can attach for formula using Extract Textual Annotations and the indicator will update to green. If a structure is indicated as *Red* it is either incorrect or it may be a generic structure which needs to be expand. We need to correct such cases manually.

Accession	Abbreviat...	Name	Generic	Crossreference	Molecular...	Validity	F
M_10fthf_c		10-Formyltetrahydrofol...	No	C00234	C20H21N...	!	
M_12dgr...		1-2-Diacyl-sn-glycerol-...	No	C00641	C27H52O5	✖	
M_12dgr...		1-2-Diacyl-sn-glycerol-...	No	C00641	C31H60O5	✖	
M_12dgr...		1-2-Diacyl-sn-glycerol-...	No	C00641	C31H56O5	✖	
M_12dgr...		1-2-Diacyl-sn-glycerol-...	No	C00641	C35H68O5	✖	
M_12dgr...		1-2-Diacyl-sn-glycerol-...	No			?	
M_12dgr...		1-2-Diacyl-sn-glycerol-...	No			?	
M_12dgr...		1-2-Diacyl-sn-glycerol-...	No		formula added	?	
M_12dgr...		Membrane 1,2-diacylgly...	No			?	
M_12ppd...		R-Propane-1-2-diol	No	CHEBI:28972	C3H8O2	✔	
M_12ppd...		S-Propane-1-2-diol	No	CHEBI:29002	C3H8O2	✔	
M_13dpg_c		3-Phospho-D-glyceroyl...	No	CHEBI:57604	C3H4O10...	✔	
M_14gluc...		1-4-alpha-D-glucan	No	CHEBI:15444	C36H62O...	✖	
M_15dap_c		1-5-Diaminopentane	No			?	
M_1agpe...		1-Acyl-sn-glycero-3-p...	No			?	
M_1agpe...		1-Acyl-sn-glycero-3-p...	No			?	
M_1agpe...		1-Acyl-sn-glycero-3-p...	No			?	
M_1agpe...		1-Acyl-sn-glycero-3-p...	No			?	
M_1agpe...		1-Acyl-sn-glycero-3-p...	No			?	
M_1agpe...		1-Acyl-sn-glycero-3-p...	No			?	

Manually assigning chemical structure

prev|top|next

You may have noticed that metabolites in rows 2-5 all had the same cross-reference (`C00641`). These four metabolites are all *glycerolipids*. Typically chemical databases will only provide a generic form of lipids, this is the reason while four different metabolites all have the same cross-reference.

To fully capture the chemistry of these metabolites (and the reactions they participate in) we need to completely specify their structure. We can interrupt what the chemical structure should look like from their name. The `didodeca` in the first name means `2 x 12` or `2` chains of length `12`. In this case it is even easier as the suffix (`C120`) encodes the length of the aliphatic chain (`C12 = 12`) and, with the last digit, whether there is a double bond in that chain (`0` = no double bonds, `1` = a double bond). The full name can be used to determine where the double bond is located and also what stereo conformation it is. In the name `1-2-Diacyl-sn-glycerol-ditetradec-7-enoyl` the `7` indicates the double bond is between the 7th and 8th carbon of the chain, there is no stereo-conformation specified. The table below summarises the length of chains for these four lipids and their IUPAC International Chemical Identifier (InChI). The InChI line notation provides a concise representation of the chemical structure which Metingear can interpret.

Metabolite Name	Chain length	Saturated	InChI
1-2-Diacyl-sn-glycerol-didodecanoyl-n-C120	12	Yes	InChI=1S/C27H52O5/c1-3-5-7-9-11-13-15-17-19-21-26(29)31-24-25(23-28)32-27(30)22-20-18-16-14-12-10-8-6-4-2/h25,28H,3-24H2,1-2H3
1-2-Diacyl-sn-glycerol-ditetradecanoyl-n-C140	14	Yes	InChI=1S/C31H60O5/c1-3-5-7-9-11-13-15-17-19-21-23-25-30(33)35-28-29(27-32)36-31(34)26-24-22-20-18-16-14-12-10-8-6-4-2/h29,32H,3-28H2,1-2H3

1-2-Diacyl-sn-glycerol-ditetradec-7-enoyl-n-C141	14	No	InChI=1S/C31H56O5/c1-3-5-7-9-11-13-15-17-19-21-23-25-30(33)35-28-29(27-32)36-31(34)26-24-22-20-18-16-14-12-10-8-6-4-2/h11,13-14,16,29,32H,3-10,12,15,17-28H2,1-2H3/b13-11-,16-14-
1-2-Diacyl-sn-glycerol-dihexadecanoyl-n-C160	16	Yes	InChI=1S/C35H68O5/c1-3-5-7-9-11-13-15-17-19-21-23-25-27-29-34(37)39-32-33(31-36)40-35(38)30-28-26-24-22-20-18-16-14-12-10-8-6-4-2/h33,36H,3-32H2,1-2H3

We can attach these chemical structures to the metabolites in a variety of ways. The easiest way is to use the `Curate Metabolite` dialog to assign the structures in batch. If multiple structures are selected the dialog will automatically open for the next structure once the previous metabolite has been curated.

With the table sorted by accession, select the four entries in the metabolites table from rows 2 to 5.

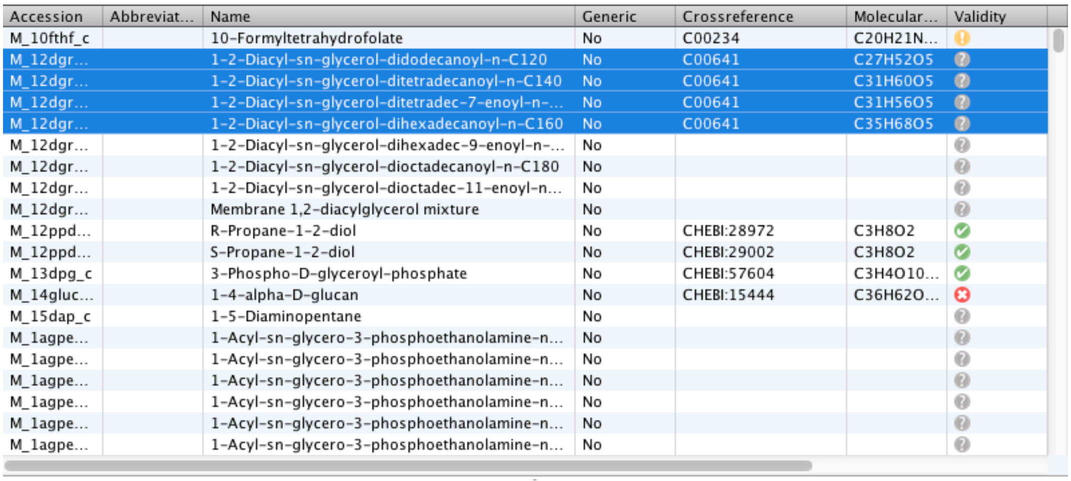

Accession	Abbreviat...	Name	Generic	Crossreference	Molecular...	Validity
M_10fthf_c		10-Formyltetrahydrofolate	No	C00234	C20H21N...	
M_12dgr...		1-2-Diacyl-sn-glycerol-didodecanoyl-n-C120	No	C00641	C27H52O5	
M_12dgr...		1-2-Diacyl-sn-glycerol-ditetradecanoyl-n-C140	No	C00641	C31H60O5	
M_12dgr...		1-2-Diacyl-sn-glycerol-ditetradec-7-enoyl-n-...	No	C00641	C31H56O5	
M_12dgr...		1-2-Diacyl-sn-glycerol-dihexadecanoyl-n-C160	No	C00641	C35H68O5	
M_12dgr...		1-2-Diacyl-sn-glycerol-dihexadec-9-enoyl-n-...	No			
M_12dgr...		1-2-Diacyl-sn-glycerol-dioctadecanoyl-n-C180	No			
M_12dgr...		1-2-Diacyl-sn-glycerol-dioctadec-11-enoyl-n...	No			
M_12dgr...		Membrane 1,2-diacylglycerol mixture	No			
M_12ppd...		R-Propane-1-2-diol	No	CHEBI:28972	C3H8O2	
M_12ppd...		S-Propane-1-2-diol	No	CHEBI:29002	C3H8O2	
M_13dpg_c		3-Phospho-D-glyceroyl-phosphate	No	CHEBI:57604	C3H4O10...	
M_14gluc...		1-4-alpha-D-glucan	No	CHEBI:15444	C36H62O...	
M_15dap_c		1-5-Diaminopentane	No			
M_1agpe...		1-Acyl-sn-glycero-3-phosphoethanolamine-n...	No			
M_1agpe...		1-Acyl-sn-glycero-3-phosphoethanolamine-n...	No			
M_1agpe...		1-Acyl-sn-glycero-3-phosphoethanolamine-n...	No			
M_1agpe...		1-Acyl-sn-glycero-3-phosphoethanolamine-n...	No			
M_1agpe...		1-Acyl-sn-glycero-3-phosphoethanolamine-n...	No			
M_1agpe...		1-Acyl-sn-glycero-3-phosphoethanolamine-n...	No			

Open the `Curate Metabolite` dialog again by choosing `Tools > Annotation > Curate Metabolite` from the menu. With the dialog open expand the `Assign Structure` section

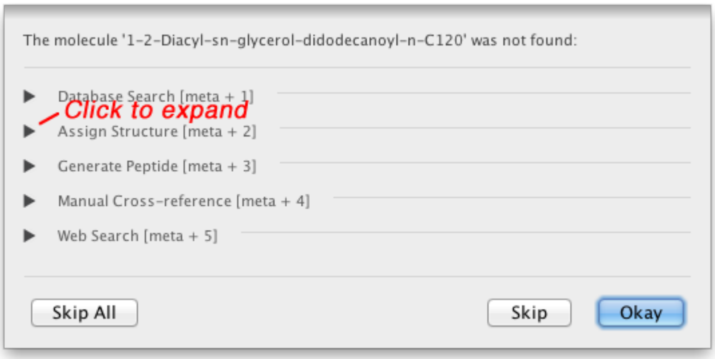

We can identify which entry we are curating by the title of the dialog.

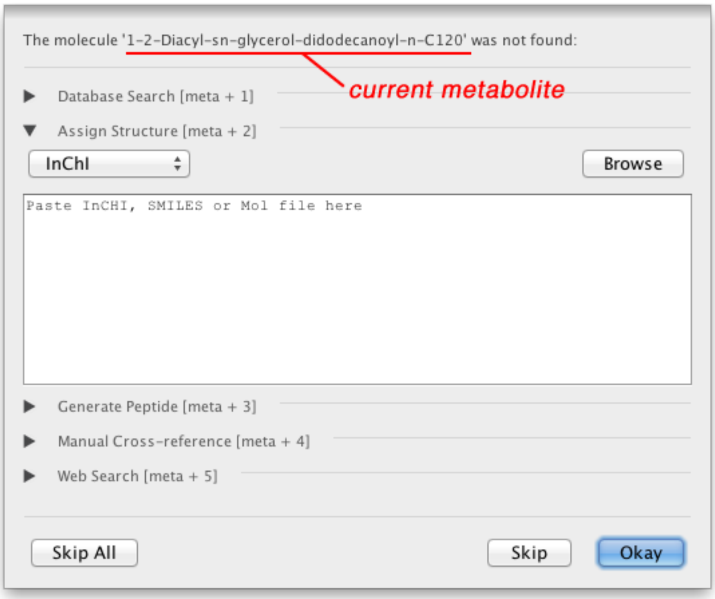

This metabolite was in our second row in the Metingear table view (first of our selection). To assign the InChI simply paste the InChI string (`InChI=1S/C27H52O5/c1-3-5-7-9-11-13-15-17-19-21-26(29)31-24-25(23-28)32-27(30)22-20-18-16-14-12-10-8-6-4-2/h25,28H,3-24H2,1-2H3`) into the text area and click `Okay` to move to the next metabolite.

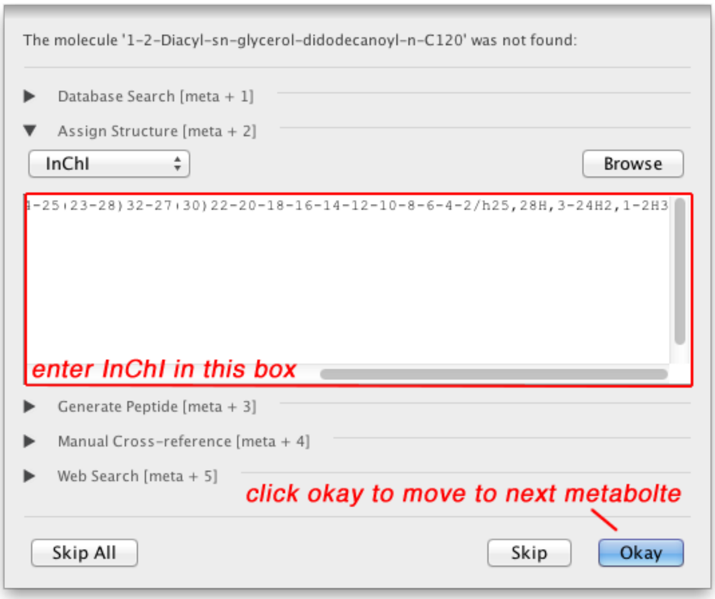

The dialog will move to the next metabolite. Again, we can identify which metabolite we are curating by the name at the top. This metabolites was in the third row of our Metingear table view (second of our selection).

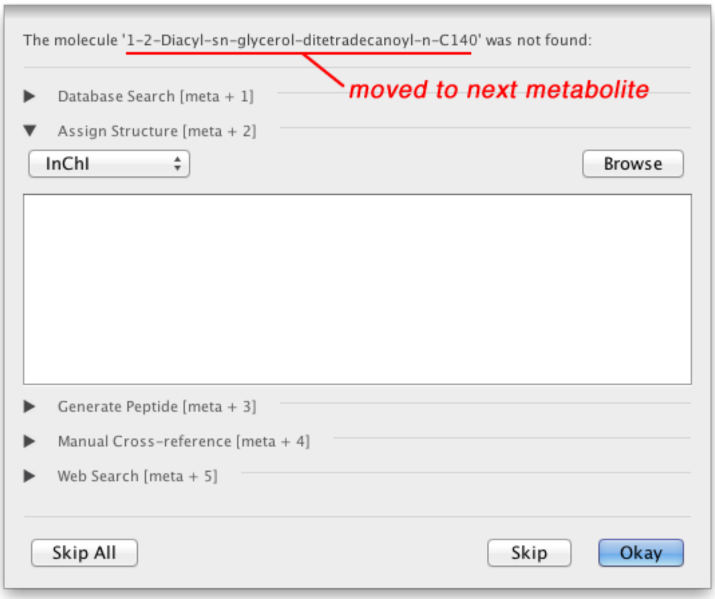

Continue to add all four InChIs, when `Okay` is clicked for the forth time the dialog will close and the metabolites will now have the InChI assigned. Click here to go back to the InChI Table.

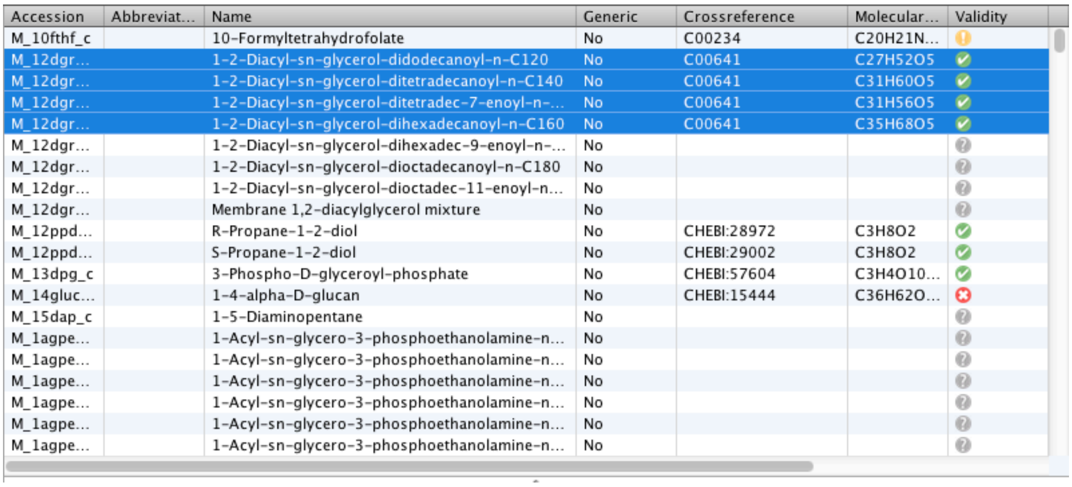

Accession	Abbreviat...	Name	Generic	Crossreference	Molecular...	Validity
M_10fthf_c		10-Formyltetrahydrofolate	No	C00234	C20H21N...	
M_12dgr...		1-2-Diacyl-sn-glycerol-didodecanoyl-n-C120	No	C00641	C27H52O5	
M_12dgr...		1-2-Diacyl-sn-glycerol-ditetradecanoyl-n-C140	No	C00641	C31H60O5	
M_12dgr...		1-2-Diacyl-sn-glycerol-ditetradec-7-enoyl-n-...	No	C00641	C31H56O5	
M_12dgr...		1-2-Diacyl-sn-glycerol-dihexadecanoyl-n-C160	No	C00641	C35H68O5	
M_12dgr...		1-2-Diacyl-sn-glycerol-dihexadec-9-enoyl-n-...	No			
M_12dgr...		1-2-Diacyl-sn-glycerol-dioctadecanoyl-n-C180	No			
M_12dgr...		1-2-Diacyl-sn-glycerol-dioctadec-11-enoyl-n...	No			
M_12dgr...		Membrane 1,2-diacylglycerol mixture	No			
M_12ppd...		R-Propane-1-2-diol	No	CHEBI:28972	C3H8O2	
M_12ppd...		S-Propane-1-2-diol	No	CHEBI:29002	C3H8O2	
M_13dpg_c		3-Phospho-D-glyceroyl-phosphate	No	CHEBI:57604	C3H4O10...	
M_14gluc...		1-4-alpha-D-glucan	No	CHEBI:15444	C36H62O...	
M_15dap_c		1-5-Diaminopentane	No			
M_1agpe...		1-Acyl-sn-glycero-3-phosphoethanolamine-n...	No			
M_1agpe...		1-Acyl-sn-glycero-3-phosphoethanolamine-n...	No			
M_1agpe...		1-Acyl-sn-glycero-3-phosphoethanolamine-n...	No			
M_1agpe...		1-Acyl-sn-glycero-3-phosphoethanolamine-n...	No			
M_1agpe...		1-Acyl-sn-glycero-3-phosphoethanolamine-n...	No			
M_1agpe...		1-Acyl-sn-glycero-3-phosphoethanolamine-n...	No			

As the InChI does not provide the coordinates of atoms the metabolite will not be updated with a chemical structure diagram. To add a structure diagram please refer to Generate Structure Diagram.

Generating chemical structure (optional)

prev|top|next

This section will demonstrate how we can generate certain chemical structures from a name. Metabolic networks can often contain reactions involving peptides. Reconstructions which model peptidoglycan synthesis will have multiple di-peptides listed in in their metabolites. Metingear can generate chemical structures for peptides by manually specifying the residues or inferring the residue sequence from the name.

To begin, use the search box and type `cys` to locate the `Cys-Gly` metabolite. With the metabolite located, double click the row in the search results to return to the entry in the `Metabolites` view.

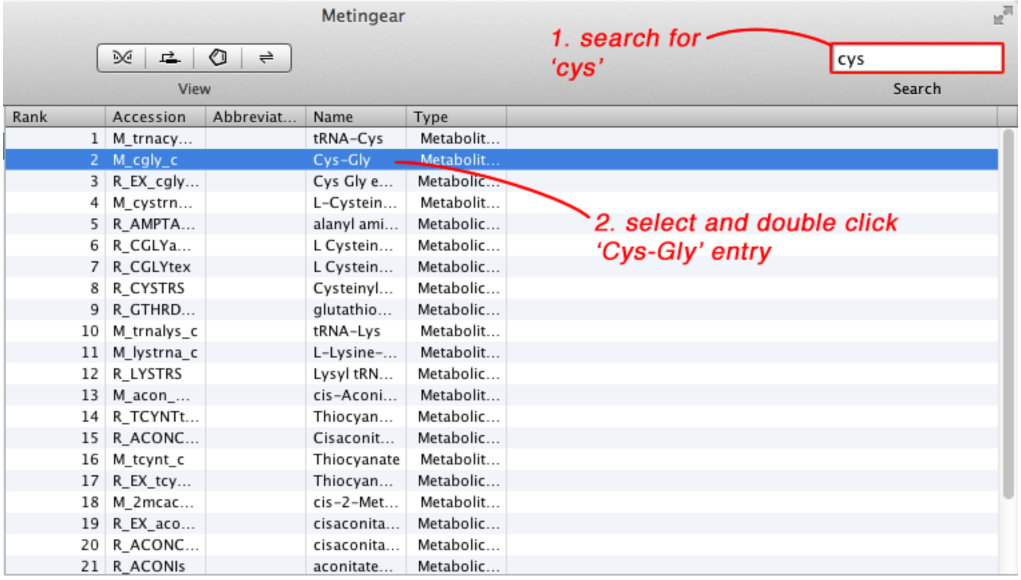

Ensuring `Cyc-Gly` is selected in the `Metabolites` view -

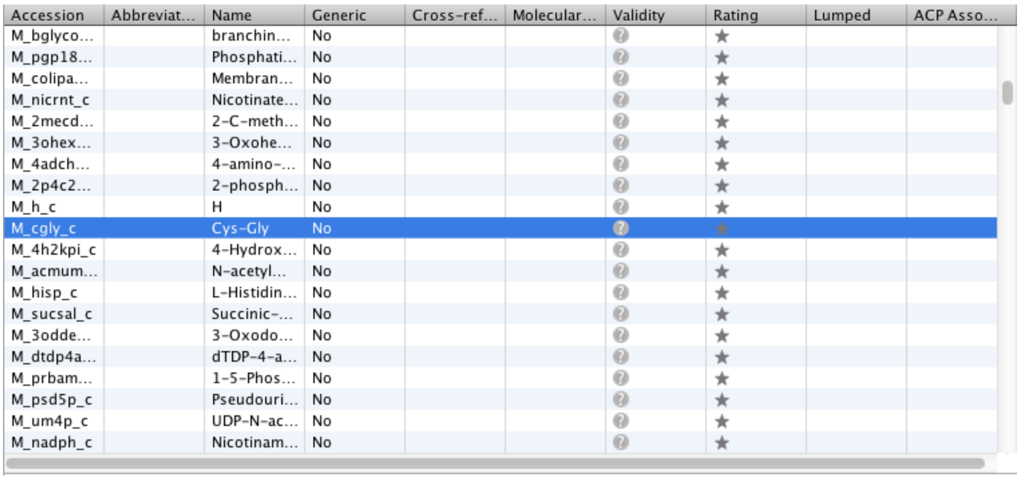

- choose the `Tools > Annotation > Curate Metabolite` menu item.

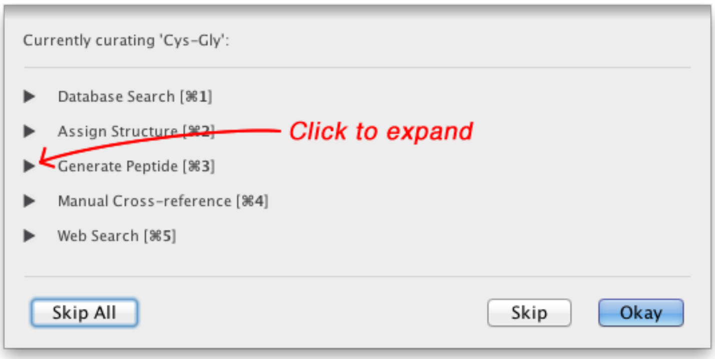

Expanding the `Generate Peptide` section will show to combination boxes. If the name of your metabolite looks like a polypeptide, Metingear will have already selected the appropriate values. When no stereo chemistry is specified the `L` form will have been chosen. You may change the residues by selecting a different value in the combination box. The chain can be lengthened or shortened using the plus (+) and minus (-) buttons.

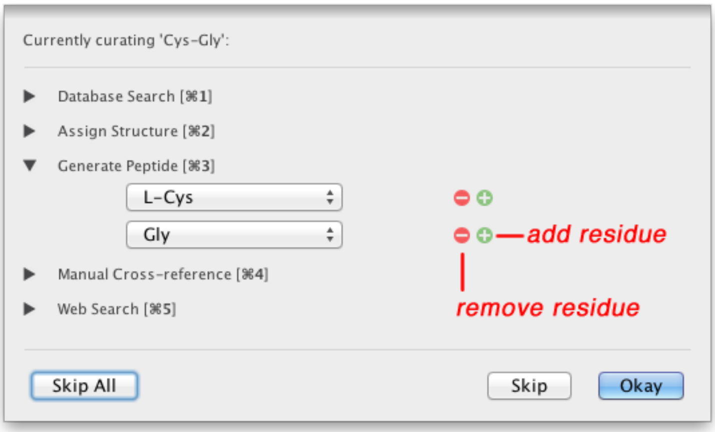

With the correct residues specified, clicking okay will assembly the peptide chain structure and attach it to the `Cys-Gly` metabolite.

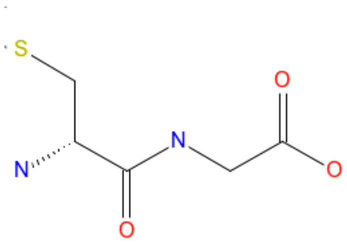

Export as annotated SBML

prev|top

The annotated metabolites (and the reactions they participate in) can be exported as SBML. The cross-references and chemical structure are specified using Resource Description Framework (RDF).

To export the active reconstruction, select the menu item `File > Export SBML`. Choose the location and the name of your SBML file (e.g. `salty-annotated.xml`) and click okay.

Here is the species output for the metabolite `R-Propane-1-2-diol` which we annotated above.

```
<species id="M_12ppd_R_e_e" name="R-Propane-1-2-diol" metaid="_000000023" compartment="e">
  <annotation>
    <rdf:RDF xmlns:rdf="http://www.w3.org/1999/02/22-rdf-syntax-ns#" xmlns:bqbiol="http://biomodels.net/biology-qu
      <rdf:Description rdf:about="#_000000023">
        <bqbiol:is>
          <rdf:Bag>
            <rdf:li rdf:resource="http://identifiers.org/obo.chebi/CHEBI:28972/"/>
            <rdf:li rdf:resource="http://rdf.openmolecules.net/?InChI=1S/C3H8O2/c1-3(5)2-4/h3-5H,2H2,1H3/t3-/m1/s1
          </rdf:Bag>
        </bqbiol:is>
      </rdf:Description>
    </rdf:RDF>
  </annotation>
</species>
```

References

- Thiele *et. al* . A community effort towards a knowledge-base and mathematical model of the human pathogen Salmonella Typhimurium LT2. *BMC Systems Biology* 2011, **5**:8 link
